# Non-Invasive Genetic Mark-Recapture as a Means to Study Population Sizes and Marking Behaviour of the Elusive Eurasian Otter (*Lutra lutra*)

**DOI:** 10.1371/journal.pone.0125684

**Published:** 2015-05-14

**Authors:** Simone Lampa, Jean-Baptiste Mihoub, Bernd Gruber, Reinhard Klenke, Klaus Henle

**Affiliations:** 1 UFZ—Helmholtz Centre for Environmental Research, Department of Conservation Biology, Permoserstrasse 15, 04318 Leipzig, Germany; 2 Friedrich Schiller University Jena, Institute of Ecology, Dornburger Strasse 159, 07743 Jena, Germany; 3 University of Canberra, Institute for Applied Ecology and Collaborative Research Network for Murray-Darling Basin Futures, ACT 2601 Canberra, Australia; Université de Sherbrooke, CANADA

## Abstract

Quantifying population status is a key objective in many ecological studies, but is often difficult to achieve for cryptic or elusive species. Here, non-invasive genetic capture-mark-recapture (CMR) methods have become a very important tool to estimate population parameters, such as population size and sex ratio. The Eurasian otter (*Lutra lutra*) is such an elusive species of management concern and is increasingly studied using faecal-based genetic sampling. For unbiased sex ratios or population size estimates, the marking behaviour of otters has to be taken into account. Using 2132 otter faeces of a wild otter population in Upper Lusatia (Saxony, Germany) collected over six years (2006–2012), we studied the marking behaviour and applied closed population CMR models accounting for genetic misidentification to estimate population sizes and sex ratios. We detected a sex difference in the marking behaviour of otters with jelly samples being more often defecated by males and placed actively exposed on frequently used marking sites. Since jelly samples are of higher DNA quality, it is important to not only concentrate on this kind of samples or marking sites and to invest in sufficiently high numbers of repetitions of non-jelly samples to ensure an unbiased sex ratio. Furthermore, otters seemed to increase marking intensity due to the handling of their spraints, hence accounting for this behavioural response could be important. We provided the first precise population size estimate with confidence intervals for Upper Lusatia (for 2012: $\hat{N}$ = 20 ± 2.1, 95% CI = 16–25) and showed that spraint densities are not a reliable index for abundances. We further demonstrated that when minks live in sympatry with otters and have comparably high densities, a non-negligible number of supposed otter samples are actually of mink origin. This could severely bias results of otter monitoring if samples are not genetically identified.

## Introduction

Elusive species play an important role in conservation. Reliable information of population status and trends are crucial for improving conservation practices and management and for addressing conservation challenges, such as antagonistic interactions between protection and conflict mitigations for species involved in human-wildlife conflicts. However, elusive species are difficult to study and therefore we often lack important demographic information. The Eurasian otter (*Lutra lutra*) is such an elusive, conflict-laden species that has suffered dramatic declines in Europe due to hunting and man-made changes to its aquatic habitats (e.g. canalisation, water pollution, prey decline) [1, 2]. Nowadays, otters benefit from protective legislations throughout Europe, and since the 1990s otter densities increased including recolonisations of areas from which they were extirpated. Since their main prey is fish, the species’ recovery inevitably resulted in conflicts with fishermen [2]. Yet, important information, such as accurate estimates of population sizes, for reconciling species conservation and human interests are still lacking in most areas of Europe [1].

Otters are elusive, mainly nocturnal, and difficult to (live-)trap [1]. They are typically monitored either by direct but mostly occasional visual counts [3, 4] or indirect records through dens [5], tracks [4, 6, 7], or faeces [8, 9]. Otter faeces (spraints) are particularly suitable to study the species, because they are used for intraspecific communication. Otters produce up to 30 spraints daily and usually defecate on frequently visited conspicuous terrestrial sites at specific locations throughout their home range (e.g. rocks, trunks, under bridges, at junctions of water channels). These marking sites and spraints can be easily detected by collectors and therefore became the “standard survey method” [8] for mapping otter distributions but also for obtaining rough estimates of population sizes (see [4] for a review).

There are contrasting opinions whether spraint counts can be used as an index of abundance. Lanszki et al. [10] found a positive correlation between relative spraint density and relative numbers of otter genotypes in an area and concluded that spraint counts are suitable as such an index. Similarly, Guter et al. [11] found a positive correlation between number of spraints and number of otter visits in latrines. However, Calzada et al. [12] criticised their study because they were not able to distinguish between individuals and could hence not tell whether all visits and spraint samples were deposited by a single individual. Other researchers have also advised against the use of spraint density as an index of population sizes because of temporal, spatial, and individual sprainting variations [4, 13, 14].

In recent studies, researchers used otter spraints for non-invasive genetic capture-mark-recapture (CMR) analyses to estimate population size [15–17].With genetic techniques, such as microsatellite genotyping, it is possible to individually identify the originator and to use this information in capture-mark-recapture (CMR) models. Non-invasive genetic CMR has become a very powerful tool since its first application in the 1990s [18, 19] to study rare and elusive species without direct handling [20, 21]. The basic principle of this approach is that non-invasively collected samples (e.g. faeces) are genotyped at multiple molecular loci (e.g. microsatellites). This multilocus genotype is then treated as a molecular individual mark. Matching genotypes are considered to belong to the same individual and are classified as recaptures. Non-matching genotypes indicate newly captured animals. Hence, for each sampling occasion, all individuals are determined to be either captured (coded as 1) or not captured (coded as 0), resulting in individual capture histories that are used for CMR analyses. Non-invasive genetic CMR opens up the possibility to obtain estimates of population size, sex ratio, survival, migration, fecundity, or population growth [21].

However, there are several difficulties that must be overcome, such as low success rates and genotyping errors [22, 23]. Genotyping errors can either result in erroneously assigning a sample to a wrong individual, because they appear to have the same genotype, or can create new so far unknown but “false individuals” (ghost individuals) by only one single loci being mistyped. The latter is more likely and can lead to overestimated population sizes [23, 24]. Several methods are available to reduce genotyping errors. We have reviewed these methods in a previous publication [23] and demonstrated that the use of an error-incorporating population size estimator is crucial to receive reliable estimates. For unbiased estimates it is also required that all individuals have a reasonable chance of being collected [23]. Hence, when using faeces as DNA source, the marking behaviour of the target species has to be understood well to avoid biased results through marking differences in e.g. sex, age, social, or reproductive status [20].

Otters are assumed to defecate in nearly equal rates regardless of their sex, reproductive status, or age [2]. However, most studies employing non-invasive genetic sampling found a male bias in their sampling [15–17, 25–28], although otter populations are likely to be slightly female-biased [1, 29, 30]. Therefore, Bonesi et al. [16] queried whether non-invasive sampling is appropriate to estimate population size and sex ratio of otters. They suggested differences in marking behaviour according to sex, social, or reproductive status as possible reasons and encouraged further research on these issues. Furthermore, Brzezinski and Romanowski [31] found that the sprainting intensity increases when spraints are previously removed. This raises the question, whether non-invasive genetic CMR is also affected by such a reaction. Both, differences in marking behaviour and changes in sprainting intensity could violate fundamental assumptions of CMR analyses.

Here, we wanted to first investigate the marking behaviour of otters by testing whether there are sex differences and to understand in which way this may influence the results of non-invasive CMR studies. Otter spraints can either be food remains or a jelly-like substance from the intestine, both with or without anal gland secretions [1, 32, 33]. Since jelly samples have higher genotyping success rates [34, 35], it should be tested whether they are defecated at equal rates by both sexes, in order to assess potential sources of bias in population size estimates. Moreover, the spraint size, level of exposure, and the marking site use might also differ between sexes, affecting spraint visibility and thus detection rate. Hence, we investigated the characteristics and intensity of spraint deposition and effects of sex-specific marking behaviour using the results of a faecal-based non-invasive genetic CMR study on a wild otter population in Eastern Saxony, Germany, over a period of six years. We estimated yearly population sizes and sex ratios demonstrating that the use of an estimator incorporating both, genotyping errors and differences in marking behaviour, is crucial to obtain reliable results. Using these population size estimates, we examined whether spraint densities could serve as an index for otter abundances by testing whether spraint densities are correlated with number of genotypes or estimated population sizes. Otter monitoring based on otter spraint counts (without genetic identification) can be hampered by samples of other sympatric living species with similar-looking faeces, such as the invasive American mink (Neovison vison). Since the mink inhabited the same area, we tested whether all samples collected as otter spraints actually derive from otters.

## Methods

### Ethics Statement

The field sampling did not involve capturing or handling of the protected otters. Therefore, we did not require permits or approvals. The accessed land is private and required permission from the fish farmers, although the pond areas are commonly used by the local population for walks or as passage.

### Study Area

The study area is located in the Upper Lusatian heath and pond landscape in the eastern part of Saxony, Germany (51°20′N, 14°19′E). Upper Lusatia covers about 5000 ha of ponds [36]. The tradition to build ponds and to use them for fish farming started already in the 13^th^ century [37]. Fish are harvested each autumn and ponds are drained. Three-year-old fish are sold, whereas spawning and young fish (1–2 years) are reinserted to smaller and deeper wintering ponds. In spring, summer ponds are filled with water again and stocked with fish. Besides the commercial function, the ponds offer an important habitat for many endangered species, such as the Eurasian otter. Due to fish production, the Upper Lusatia is believed to host one of the biggest and most viable otter populations in Central Europe [29, 38, 39].

**Fig 1:**
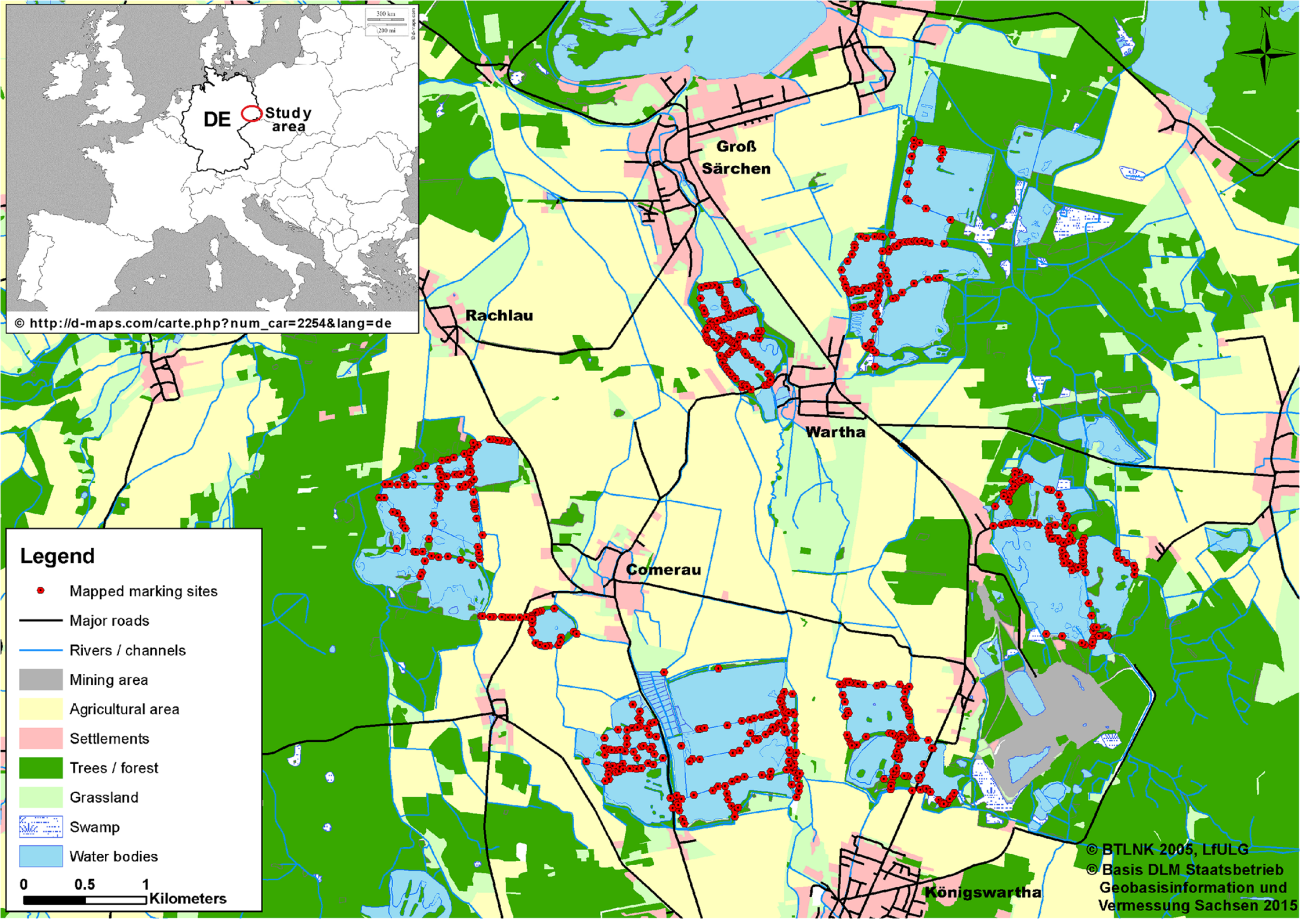
Study area map with recorded otter marking sites. Location of otter marking sites (red hexagons) in seven pond areas and one single pond in the Upper Lusatia (Saxony, Germany), where we searched for fresh faeces for genetic capture-mark-recapture (CMR) analyses (2006–2012). Main land use types of the surrounding area are outlined.

The study area included 64 ponds (505 ha total water surface) that are clustered in seven pond areas, each comprising 8–13 ponds of varying size (0.36–39.6 ha) (Fig 1). All ponds are connected by a complex system of ditches and streams and framed by naturally vegetated embankments that are partly used as agricultural roads. Islands, extensive reed belts, and heavily vegetated peninsulas can serve as resting sites for otters and induce heterogeneous structures. The pond areas are surrounded by pasture, cropland, forest, roads and urban areas.

The inset of Europe is reprinted from “http://d-maps.com/carte.php?num_car=2254&lang=de" under a CC BY license, with permission from "d-maps.com", original copyright 2007 d-maps.com. Land use types are reprinted from “BTLNK 2005” under a CC BY license, with permission from "Sächsisches Landesamtes für Umwelt, Landwirtschaft und Geologie (LfULG)", original copyright 2005 LfULG. Roads and rivers are reprinted from “ATKIS-Basis DLM data” under a CC BY license, with permission from “Staatsbetrieb Geobasisinformation und Vermessung Sachsen”, original copyright 2015 GeoSN.

### Sampling and Microsatellite Genotyping

From 2006 to 2012 (except 2009, missing sampling year), faecal collection was done on five consecutive days just before (March 2006, 2010, 2011, 2012) or just after (April 2007, May 2008) fish relocation into summer ponds. The chosen sampling months (March–May) are considered to be off-peak seasons for otter reproduction in Eastern Germany [40].

In each year, all ponds filled with water were included in the sampling. The number of ponds varied over years, due to the seasonally and yearly differing water regime management (Table 1). Each annual faecal collection started with a pre-sampling day on which we recorded active otter marking sites and marked already dropped spraints to facilitate recognition of fresh spraints the next day. In the morning of the following five days, all freshly deposited samples were collected from known or newly discovered marking sites. For each sample, we recorded location of marking site, size category of sample (small [≤ 1.5 cm], medium [≤ 3 cm], large [> 3 cm]), its degree of sliminess (spraint, spraint plus mucus, jelly), its exposure level (actively exposed (e.g. scratch piles), passively exposed (e.g. stones, roots, sticks, grass tussock), or non-exposed), and total number of old/fresh samples found on the marking site (1–2, 3–4, > 4). For each fresh spraint, the external layer containing sloughed gut cells was wiped off with a cotton stick. Cotton sticks were placed in a separate sterile 10 ml cryovial (Biozym Scientific, Hessisch Oldendorf, Germany) and either extracted on the day of collection (year 2006) or stored at—80°C until extraction in 1.8 ml buffer ASL (Qiagen, Hilden, Germany) (years 2007–2012).

**Table 1:** Results of faecal-based genetic CMR samplings (2006–2012) from a wild otter population living in pond areas in Upper Lusatia (Saxony, Germany).

Sampling year	Sampling period	Water surface (ha)	Active marking sites	Collected samples	Extracted samples	Sure otter samples	Sure mink samples	Other species	Unknown samples	Genotyped samples	Individual genotypes
2006	27–31 Mar	261	130	356	257	199	7	0	51	121	22
2007	23–27 Apr	399	92	282	270	211	7	0	52	134	30
2008	26–30 May	449	87	198	196	136	7	1	52	96	22
2010	22–26 Mar	294	172	381	367	204	50	3	110	130	21
2011	28 Mar–1 Apr	366	173	461	459	239	57	7	156	138	26
2012	27–31 Mar	360	159	454	452	284	33	7	128	159	24
**Total**		**505**	**384**	**2132**	**2001**	**1273**	**161**	**18**	**549**	**778**	**84**
**Mean**				**355.3**	**333.5**	**212.2**	**26.8**	**3**	**91.5**	**129.7**	**24.2**

Water surface: sum of all ponds filled with water. Sure otter samples: samples for which we recorded at least one expected otter allele. Sure mink samples: samples that were identified as mink. Unknown samples: samples that did not produce any PCR product. Genotyped samples: samples successfully genotyped on seven microsatellites and successfully sexed.

DNA was extracted from all samples employing the QIAamp DNA Stool Mini Kit (Qiagen) and stored afterwards at—20°C (for details see S1 Supporting Information). We followed all precautions recommended by Lampa et al. [23] to rigorously prevent cross-contamination during extraction and amplification. Extracted samples were genotyped using seven microsatellite markers (Lut435, Lut457, Lut604, Lut615, Lut701, Lut733, Lut914) [41–43] and sexed with markers Lut-SRY [43] and DBY7Ggu [44]. The latter was designed for wolverines (*Gulo gulo*) but also amplifies in male otters [44, 45]. The nine loci were multiplexed in three primer sets (M1: Lut 457, 615, 733; M2: Lut 435, 604, 701; M3: Lut 914, SRY, DBY7Ggu) (see S1 Supporting Information for details on PCR conditions).

Because otter faecal samples from our study area have fairly high genotyping error rates and low genotyping success rates [23, 34], the genotypes after one PCR per locus contained too many errors. Hence, it was crucial to repeat amplifications generating hereby a consensus genotype. To minimise costs and efforts, we followed a screening approach that consists of five amplification steps after that low-quality samples were removed according to certain thresholds (see [23] for more details). The first amplification step was also used to screen the dataset for non-target species (e.g. mink). After the fifth amplification step, all samples that generated a genotype at all but one or two loci were repeated until a reliable genotype could be assigned to the missing markers (up to 27 repeats).

The generated consensus genotypes were compared to each other; equal genotypes were scored as belonging to the same individual. Similar genotypes that mismatched at one or two alleles were re-amplified three times at the locus in question to ensure that this was not due to genotyping errors. All successfully genotyped samples were then amplified with the primer set M3 to identify sex. Individuals were identified as males after three sightings of the targeted peak. If all samples of an individual showed no PCR signal after three amplifications, we sexed this individual as a female. Individuals with less than three samples were six times amplified if no targeted peak was recorded to ensure that these samples derived from a female otter.

The six datasets of each year were subsequently checked for still extant genotyping errors with Programme DROPOUT [46] that determines probably erroneous samples (EB-test) or loci (DCH-test). Actual genotyping error rates were calculated following Broquet and Petit [47] by comparing scored genotypes with the consensus genotype (see also [23]). Amplification success rates were calculated by dividing the number of PCRs showing at least one expected allele by the number of conducted PCRs, while genotyping success rates depict the number of successfully genotyped samples relative to the number of extracted otter samples. Mean expected and observed heterozygosities (H_e_, H_o_) and sample size corrected probabilities of identity (PI), as well as PIs for siblings (PI_sib_) were computed over all six loci using software GIMLET 1.3.3 [48]. All calculations were done for each year separately and an overall mean is provided.

### Marking Behaviour

For a better understanding of the otter marking behaviour, we first assessed whether spraint sliminess, amount, and exposure, as well as marking site utilisation were different for males and females. For this purpose, we pooled all successfully genotyped samples from all years and conducted a Pearson's chi-squared test for each of the four spraint characteristics. To correct for the multiple testing problem, p-values were adjusted following the Bonferroni-Holm correction [49].

To see if males and females defecate at similar rates, we compared the number of deposited spraints per individual first over all years taking the mean number of samples per individual over the six years. Since the means were not normally distributed, we compared males and females applying the non-parametric Mann-Whitney-U-test. We further tested each year separately for sex differences by taking the actual deposited number of scats per individual, using two-sample permutation tests for integer valued observations implemented in the R-package *exactRankTests* [50]. To account for alpha error accumulation, p-values were adjusted according to Bonferroni-Holm procedure.

Furthermore, we were interested in whether the three different spraint types are more or less often placed exposed and on frequently used marking sites and whether the latter have more or less often exposed samples (e.g. to see if higher quality jelly samples are easier to find for collectors). Using Kendall rank correlation coefficients, we tested for correlations between the sliminess, exposure level, and number of spraints found on the respective marking site, respectively. For this, we pooled all samples that showed at least one expected otter allele (sure otter samples). P-values were adjusted for the three correlations following the Bonferroni-Holm procedure.

The statistics performed in this chapter are done in the R environment [51].

### Population Size Estimation

We estimated population sizes for each year using closed population CMR models [52], because these models require fewer estimated parameters, allow more flexible assumptions, and provide most precise and unbiased estimates. Closed models require that birth, death, or migration between sampling occasions is negligible. Because our study area was large and we sampled on five consecutive days outside the main reproductive period, these assumptions are very likely met, which was supported by a test in a previous publication [23].

Since it is unlikely that all genotyping errors were completely eliminated from the datasets [23], we employed the error-incorporating misidentification model from Lukacs and Burnham [53] (hereafter L&B estimator) implemented in Program MARK [54]. The L&B estimator adds to each closed population model available in MARK the misidentification parameter α—the probability of a correct classification. An α close to 1 indicates a low probability of still extant genotyping errors.

We estimated separately for each year the population size ($\hat{N}$— for males, females, and total), conditional capture (p) and recapture (c) probability, probability of a correct classification (α), and number of genotypes never captured (f_0_). We fitted a variety of models to the data that incorporated no capture variation (M_0_), individual (M_h_), behavioural M(_b_), or daily varying (M_t_) catchability and combinations thereof (M_bh_, M_th_, M_tb_). Since we observed a daily increase in the number of collected samples that peaked in the third or fourth sampling day and mostly decreased on the fifth day, we tested if this pattern was introduced by already sampled otters that displayed a daily changing recapture rate (c_1_, c_2_, c_3_, c_4_), while the probability to be newly captured (p) remained constant. Each model was fitted with and without a sex difference.

Individual heterogeneity (p_i_) is difficult to separate from misidentification (α), incorporating both can lead to inconclusive results [54]. Whenever p_i_ and α were only poorly estimable, we dropped this model from the candidate model set. Models were adjusted for correct parameter counts where confounding or boundary estimates required it.

We ranked models employing corrected Akaike’s Information Criterion (AIC_c_) that accounts for small sample sizes [55, 56]. Using normalised AIC_*c*_ weights, reflecting the likelihood of a model [57], we calculated a weighted average for all parameter estimates ($\hat{N}$_males_, $\hat{N}$_females_, $\hat{N}$_total_, p, c, α, f_0_). If supported models had unidentifiable parameters, a weighted average estimate for the unidentifiable parameter was calculated by dropping the respective model, but not for estimates of identifiable parameters. The model weighted average capture and recapture probabilities were weighted once more by the respective weighted average p_i_-value (heterogeneity parameter) and summarised for each day to receive a daily re/capture probability. With the obtained weighted average population sizes of males and females, we calculated yearly sex ratios (male to female mean) and used the total population size to compute population densities per water area (in ha), per km shoreline, and for the total area studied.

Finally, we wanted to test the hypothesis that spraint densities are good indicators for otter densities. Similar as in Lanszki et al. [10], we used a linear regression to check whether yearly numbers of genotyped scats per ha explain yearly numbers of genotyped individuals per ha or yearly numbers of estimated individuals per ha.

## Results

### Sampling and Microsatellite Genotyping

Out of 2132 collected faecal samples, 2001 were suitable for DNA extraction (Table 1). After the first three amplifications with the multiplex trio M1, 179 samples could be identified as being either from minks (*Neovison vison*) or other unknown species (Table 1). All mink samples showed much shorter PCR products than the expected otter alleles and were compared to reference mink samples from an animal park in Leipzig, Germany (mink alleles: 120bp for Lut457; 95bp for Lut615; 142/146/150/154bp for Lut733). While in years 2006–2008 we only found seven mink samples each, the number increased tremendously from 2010 on (Table 1). We were able to receive numbers of harvested minks (minks per trapnight—MPT) for one of our pond areas (100 ha) that clearly demonstrated an increase in minks for this period: MPT_2008_ = 0.028; MPT_2009_ = 0.021; MPT_2010_ = 0.091 (kindly provided by A. Lehmann).

Since further 549 samples did not produce any PCR product at all and may also belong to other species, the numbers of samples for which we recorded at least one expected otter allele decreased to 1273 (Table 1). We were able to obtain complete multilocus genotypes for 778 samples (Table 1), with a mean genotyping success rate over the years of 62.1% (57.7% (2011)– 70.6% (2008)) considering all verified otter samples. Mean amplification success rates over all years amounted to 79.9% (75.5% (2006)– 83.6% (2008)) for autosomal markers, 87.3% (83.1% (2010)– 91.8% (2012)) for the gonosomal marker Lut-SRY, and 54.2% (40.9% (2010)– 80% (2007)) for DBY7Ggu. Mean heterozygosities amounted to H_e_ = 0.51 (0.49 (2011)– 0.54 (2006)) and H_o_ = 0.6 (0.54 (2011/2012)– 0.65 (2006)). The PI ranged between 5.3 × 10^–5^ (2006) and 1.6 × 10^–4^ (2010) and the PI_sib_ between 1.6 × 10^–2^ (2006) and 2.4 × 10^–2^ (2011), which was sufficiently low.

Genotyping error rates over all years amounted to 48.9% (AD rate = 45.1% (39.3% (2012)– 48% (2006)); FA rate = 3.8% (2.9% (2006)– 4.6% (2012)). According to Programme DROPOUT [46] no locus had significantly more errors than any other and had to be dropped. However, in 2006, 2007, and 2011 artificially 4–5 new individuals were produced compared to only two new individuals in other years. Also the numbers of mismatching loci showed too many 1MM and 2MM pairs in 2006, 2007, and 2011. Hence, although we amplified each sample on average 5.6 times per loci (range 3–27), datasets are likely to still contain genotyping errors. Since further replications would probably not eradicate all errors, it was necessary to employ analysis methods that can incorporate genotyping errors.

The genotyped samples could be pooled to 79 distinct genotypes out of which five dyads (pairs) showed different sexes resulting in 84 different individuals (43 ♂, 41 ♀). Most individuals were found in only one year (27 ♂, 19 ♀), some in two (10 ♂, 11 ♀) or three (5 ♂, 6 ♀) years (not necessarily consecutive years), and few were found in four years (1 ♂, 5 ♀).

### Marking Behaviour

**Fig 2:**
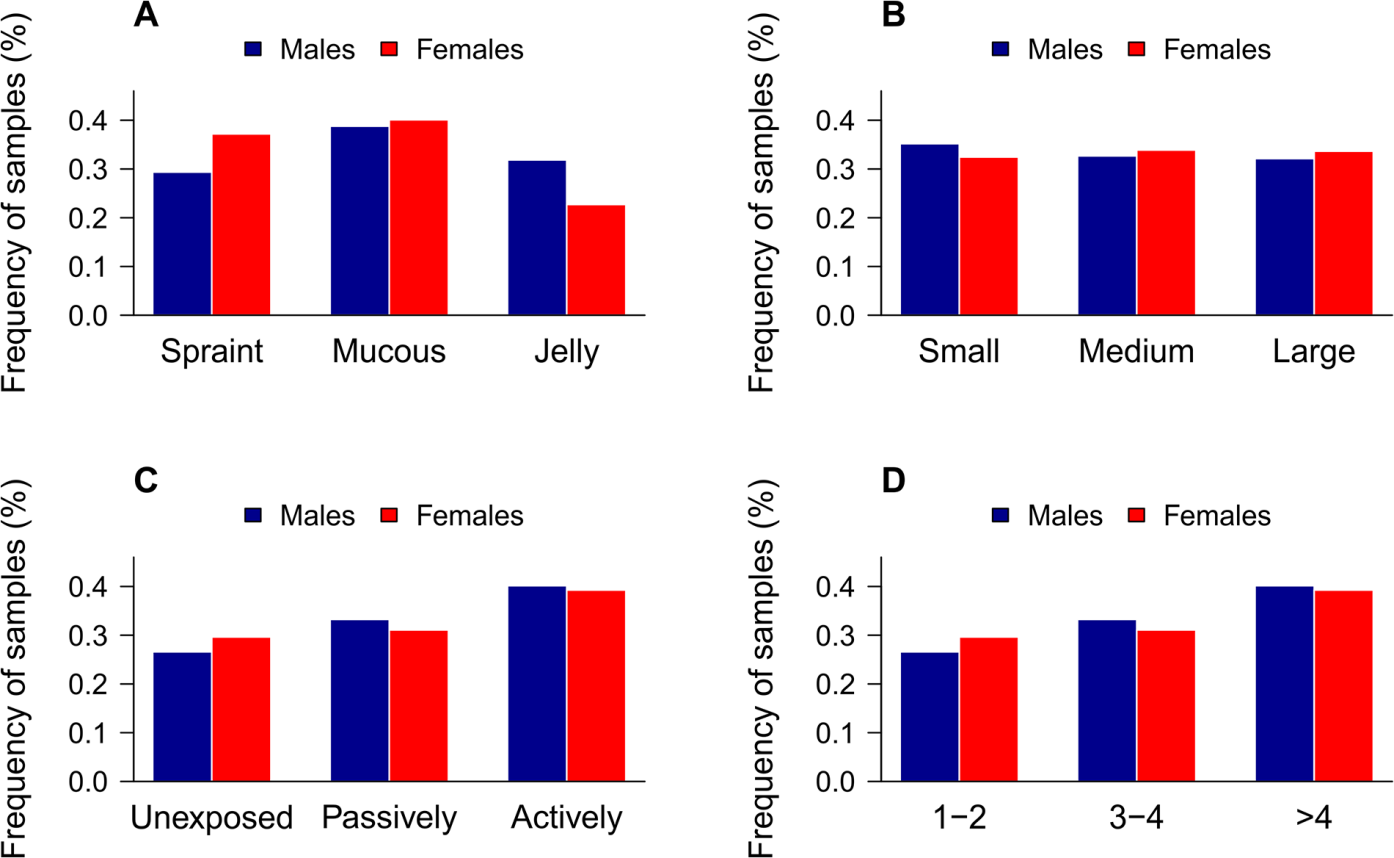
Sex differences in otter marking behaviour. Frequency of genotyped otter samples regarding (A) their sliminess (spraint, spraint plus mucus, jelly samples), (B) their size (small, medium, large), (C) their level of exposedness (non-exposed, passively exposed, actively exposed), and (D) the number of otter faeces at the specific marking site (1–2, 3–4, >4 samples); all four separated by sex. Only sliminess showed a significant sex difference in a Pearson’s chi-squared test (χ^2^ = 9.6, df = 2, p_-adjusted_ = 0.0082).

Testing size, sliminess, exposedness, and marking site utilisation for differences in sex revealed that only sliminess was significantly different between males and females (Pearson’s chi-squared test: χ^2^ = 9.6, df = 2, p_-adjusted_ = 0.0082). Males significantly defecated more often jelly samples and less often spraints than females (Fig 2).

The maximum number of scats deposited by one individual within a yearly sampling period amounted to 26. Within one night, individuals defecated on average 1.76 spraints with a maximum of 11. Both maxima were generated by males. However, taken over all years sex had no significant effect on the number of deposited scats (U-test: W = 971.5, p = 0.4189; mean_-males_ = 4.9, median_-males_ = 4, mean_-females_ = 4.7, median_-females_ = 4.5). Hence, there were also no significant differences within a year (permutation tests: p_-2006_ = 0.51; p_-2007_ = 0.46; p_-2008_ = 0.22; p_-2010_ = 0.083; p_-2011_ = 0.28; p_-2012_ = 0.97).

The correlations between sliminess and exposedness showed that the more slime a sample consists of the more often it is placed exposed (more often actively than passively), whereas less slimy spraints are more often deposited in a non-exposed way (Kendall’s tau = 0.087, z = 3.47, p-_adjusted_ = 0.0011). On marking sites that were not used the days before, we found less often jelly samples than on marking sites with at least five old/fresh spraints (Kendall’s tau = 0.063, z = 2.52, p = 0.012). When correlating the exposedness with the number of samples on a marking site, the results showed that the more samples are deposited on a marking site the more likely they are actively exposed (Kendall’s tau = 0.16, z = 6.42, p-_adjusted_ = 4.23 × 10^–10^).

### Population Size Estimation

In four years (2008–2012) we had to drop individual heterogeneity models from the candidate model set because heterogeneity was confounded with misidentification (Table 2). All models with sex-dependent parameters (p_i_, p, c, α, f_0_) showed no significant difference in a likelihood-ratio test compared to the respective model without the sex effect and were always ranked lower with ΔAIC_*c*_ between 3.9 and 29 (mean = 12.1). Thus, these models were dropped from the candidate model set. The model and p_i_ (within year capture heterogeneity) weighted average capture probabilities (p) were relatively high for each year (0.48–0.75; mean = 0.57 ± 0.07), whereas the model and p_i_ weighted average recapture probabilities (c) were even higher (0.54–0.79; mean = 0.65 ± 0.07) (Table 2). Except for year 2010, where we found equal but very high capture and recapture rates, the recapture probability was always higher than the capture probability, with differences between 0.011–0.23 (averaged difference = 0.08).

**Table 2:** Closed population models with misidentification run in Programme MARK to estimate yearly population sizes for males and females of a wild otter population in Upper Lusatia (Saxony, Germany).

		2006			2007			2008			2010			2011			2012		
Model	K	ΔAIC_c_	*w*_*i*_	$\hat{N}$ ± SE	ΔAIC_c_	*w*_*i*_	$\hat{N}$ ± SE	ΔAIC_c_	*w*_*i*_	$\hat{N}$ ± SE	ΔAIC_c_	*w*_*i*_	$\hat{N}$ ± SE	ΔAIC_c_	*w*_*i*_	$\hat{N}$ ± SE	ΔAIC_c_	*w*_*i*_	$\hat{N}$ ± SE
M_0_	3	**0.00**	**0.44**	**19 ± 2.6**	5.81	0.02	24.0 ± 3.5	6.06	0.04	19.4 ± 2.2	**0.00**	**0.49**	**15.4** ± **2.1**	**0.00**	**0.33**	**25.1** ± **1.8**	10.83	0.00	21.5 ± 2.2
M_h_	5	2.73	0.11	20 ± 2.8	**0.00**	**0.39**	**27.9** ± **5.5**	**0.00**	**0.82**	**21.9 ± 4.4**	3.62	0.08	15.5 ± 2.2	–	–	–	–	–	–
M_b_	4	0.33	0.37	18 ± 2.6	1.97	0.15	23.5 ± 4.4	6.16	0.04	18.9 ± 2.2	2.16	0.17	15.4 ± 2.1	1.26	0.17	24.6 ± 1.8	3.12	0.12	20.6 ± 2.1
M_t_	7	7.44	0.01	18.6 ± 2.7	7.38	0.01	22.7 ± 3.3	13.68	0.00	19.6 ± 2.2	2.13	0.17	15.4 ± 2.1	0.38	0.27	24.3 ± 1.7	1.41	0.27	20.4 ± 2.1
M_bh_	7	5.42	0.03	18.7 ± 2.9	3.53	0.07	27.8 ± 7.1	–	–	–	–	–	–	–	–	–	–	–	–
M_th_	13	15.85	0.00	18.9 ± 2.6	8.48	0.01	26.3 ± 6.0	–	–	–	10.36	0.00	16.4 ± 2.0	2.39	0.10	23.8 ± 1.5	–	–	–
M_tb_	11	9.82	0.00	18.5 ± 2.6	1.93	0.15	22.8 ± 3.3	4.48	0.09	19.2 ± 2	11.55	0.00	15.4 ± 2.1	7.23	0.01	24.4 ± 1.5	4.77	0.05	20.3 ± 2.1
M_tb_constrained_	7	5.16	0.03	18.5 ± 2.6	1.30	0.21	24.9 ± 4.6	9.02	0.01	19.2 ± 2	3.57	0.08	15.4 ± 2.1	1.97	0.12	24.4 ± 1.7	**0.00**	**0.56**	**20.3 ± 2.1**
$\hat{N}$_males_ ± SE (CI)		10.2 ± 1.5 (7.3–13.1)			12 ± 2.6 (7–17.1)			10.7 ± 2.1 (6.6–14.9)			6.6 ± 0.9 (4.8–8.4)			10.4 ± 0.8 (8.9–11.9)			8.2 ± 0.8 (6.5–9.8)		
$\hat{N}$_females_ ± SE (CI)		8.5 ± 1.2 (6.1–11)			13.7 ± 2.8 (8.1–19.2)			10.7 ± 2.1 (6.6–14.9)			8.8 ± 1.2 (6.4–11.2)			14.2 ± 1 (12.1–16.2)			12.2 ± 1.2 (9.8–14.7)		
$\hat{N}$_total_ ± SE (CI)		18.7 ± 2.7 (13.4–24)			25.7 ± 5.4 (15.1–36.3)			21.5 ± 4.2 (13.2–29.8)			15.4 ± 2.1 (11.2–19.6)			24.6 ± 1.8 (21–28.1)			20.4 ± 2.1 (16.3–24.5)		
p ± SE		0.5 ± 0.12			0.48 ± 0.28			0.53 ± 0.16			0.75 ± 0.09			0.59 ± 0.12			0.56 ± 0.14		
c ± SE		0.57 ± 0.13			0.62 ± 0.29			0.54 ± 0.16			0.75 ± 0.1			0.63 ± 0.12			0.79 ± 0.1		
α ± SE		0.85 ± 0.12			0.81 ± 0.14			0.93 ± 0.11			0.73 ± 0.1			0.95 ± 0.07			0.82 ± 0.08		

Numbers of modelled parameters (K), differences in AICc (ΔAICc), AICc model weights (*w*_*i*_), and estimated population sizes with standard errors ($\hat{N}$ ± SE) are shown for all models, except of models were heterogeneity and misidentification were confounded (–). Highest ranked models according to AICc are highlighted in bold. For each year, we specified the weighted average population size for males (${\hat{N}}_{\text{males}}$), females (${\hat{N}}_{\text{females}}$), and the entire population (${\hat{N}}_{\text{total}}$) together with their standard errors (SE) and 95% confidence intervals (CI). Furthermore, pi and model weighted average capture (p) and recapture (c) probabilities and model weighted misidentification parameters (α) are given with their standard error (SE).

The average misidentification parameter α ranged between 0.73 and 0.95 (mean = 0.85 ± 0.04), indicating that some samples were misidentified in each year with a probability of 5–27%. Hence, each year’s dataset still harboured ghost individuals and hence genotyping errors. The derived population size estimates ($\hat{N}$) of all models for a particular year were very similar, even for those having AIC_*c*_ weights < 0.01. The model weighted average population size using AIC_*c*_ weights for each year ranged between 15 (2010) and 26 (2011) individuals (mean = 21) (Table 2). In four years (2007/10/11/12), sex ratios ranged between 0.67 and 0.88. In 2008 the sex ratio equalled 1 and only in 2006 we found more males than females with a sex ratio of 1.2.

Using average population sizes, otter densities in our study area ranged from 0.048 (2008) to 0.072 (2006) otters per ha pond (mean = 0.06), from 0.34 (2008) to 0.48 (2006/2007) otters per km pond shore (mean = 0.42), and from 0.004 (2010) to 0.007 (2007) otter per ha area regarding the entire study area (36 km^2^) (mean = 0.0058).

**Fig 3:**
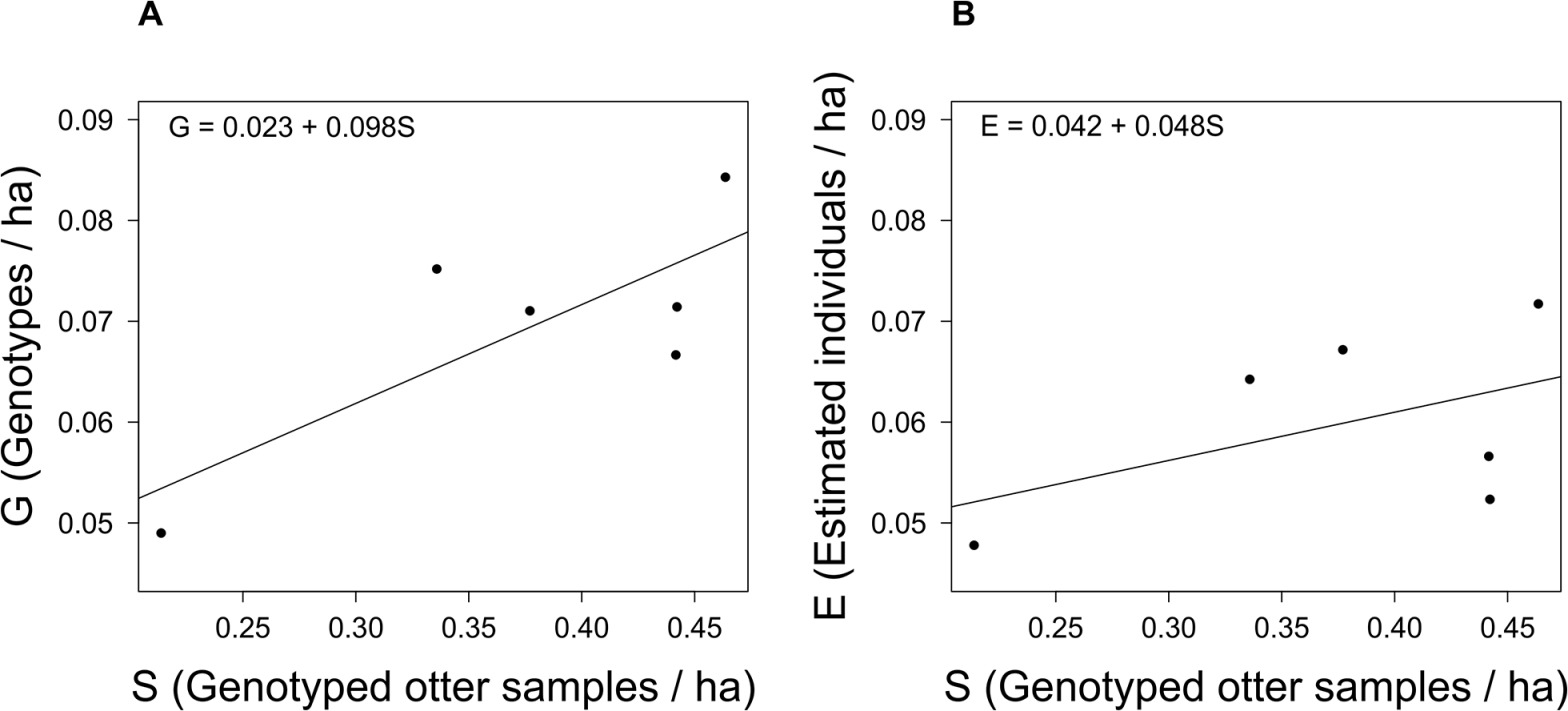
Relationship between spraint densities and otter numbers. Linear regressions between number of genotyped otter samples per ha (S) and (A) number of genotypes per ha (G) (R^2^ = 0.62, df = 4, p = 0.063) and (B) number of estimated individuals (E) (R^2^ = 0.24, df = 4, p = 0.33), respectively. Equations for both regressions are offered.

The linear regressions to test whether spraint densities are good indicators for otter densities revealed a near-significant relationship between yearly spraint densities (numbers of genotyped samples per ha) and yearly numbers of distinct genotypes per ha (R^2^ = 0.62, df = 4, p = 0.063, Fig 3), whereas there was no relationship between yearly spraint densities and yearly estimated individuals per ha (R^2^ = 0.24, df = 4, p = 0.33, Fig 3).

## Discussion

### Microsatellite Genotyping and Population Size Estimation

The genotyping error rate (GER) was quite stable over the six sampling years (range: 0.44 (2012)– 0.51 (2006)), but fairly high compared to other otter studies that used the same way of calculation (15: GER = 20.9%, 16: GER = 18.1%, 45: GER = 17.3%, 58: GER = 31.9%). While the GER represents errors that are already removed from the data, the two tests in DROPOUT and the misidentification parameter α indicated that errors might still be present in the yearly datasets. Therefore, it is crucial to use population size estimators that account for genotyping errors if they cannot be entirely removed [20, 23, 24].

One reason for these high GERs might be the comparable high number of repetitions (up to 26 times) to gain increased genotyping success rates. Because of high error rates and low genotyping success rates, we followed a rigorous protocol including various contamination preventions during extraction and amplification, a screening approach to exclude low quality samples, and the generation of consensus genotypes via high numbers of repetitions [23]. Although those steps minimised errors they could not save us from having still undetected errors in the consensus genotypes.

Although error-incorporation is crucial for estimating population sizes and sex ratios, the statistical tests implemented for understanding the marking behaviour were less sensitive to ghost individuals. That is either because individual identification was irrelevant or because we only compared males with females. Since both sexes showed no significant difference in the number of single samples—that are potential ghost individuals—and also re/capture probabilities were equal between males and females, the number of ghost individuals should be evenly distributed among sexes. Thus, we regard the results of the tests for marking behaviour as trustworthy.

Despite acceptable low PIs [23], we had five dyads that had identical autosomal genotypes but different sexes. In two cases both individuals of the dyad were either found dead subsequently, were collected in several years, or were represented by a high number of samples within a year (≥ 9); they are hence likely to exist and to be closely related (e.g. siblings). For the remaining three dyads, one sex (2 ♂, 1 ♀) was only represented by a single sample in a given year and could thus be an erroneously sexed sample. Since further repetitions could not prove this and since it applied to both sexes, we treated the found genotypes to be real ones.

In each year, we had one to six more genotyped than estimated individuals. If the actual number was not underestimated, we captured most resident individuals, which can be explained by the high sampling intensity. Most studies estimating otter densities were conducted at rivers, streams, or ditches [10, 30, 58–60], some at lakes or coasts [1, 25, 61], but only a few in fish pond landscapes [15, 27] (Table 3). While densities seem to be lower at rivers and lakes than in fish pond landscapes (Table 3), one needs to bear in mind that comparability is limited because of different methods and water body shapes. Two studies, that also investigated fish pond landscapes employing non-invasive genetic methods, obtained higher estimates per total area [15] or per km pondside [27] (Table 3). Besides differences in pond sizes and overall landscape structures, methodological reasons could also account for this difference, because neither Hajkova et al. [15] nor Lanszki et al. [27] accounted for genotyping errors. The former used an estimation method, CAPWIRE [62], that does not account for genotyping errors. The latter counted the number of genotypes without employing population size estimators. If we would have used the same approaches, our densities would have been larger and comparable to both studies (0.006–0.009 otter per ha area using CAPWIRE; 0.35–0.56 otter per km pond shore using number of genotypes).

**Table 3:** Otter densities of different studies including information on studied habitats and on employed methods.

Study	Otter per ha area	Otter per ha water area	Otter per km shoreline	Habitat studied	Method used
**This study**	**0.004–0.007**	**0.048–0.072**	**0.34–0.48**	**Fish ponds**	**Non-invasive genetic CMR**
Ansorge [39]	0.001–0.0013 (adults)			Fish ponds	Expert knowledge
Erlinge [61]		0.007–0.01	0.33–0.5 ^1^; 0.2 ^2^	Lakes ^1^, rivers ^2^	Tracking footprints and spraints
Hajkova et al. [15]	0.0076–0.0081^1^		0.22–0.26^2^	Fish ponds^1^, rivers^2^	Non-invasive genetic CMR
Hung et al. [58]			1.5–1.8	Rivers	Non-invasive microsatellite genotyping (MNA)
Kalz et al. [25]	0.0016	0.013	0.21	Lakes, rivers	Non-invasive microsatellite genotyping (MNA)
Koelewijn et al. [45] (reintroduced population)	0.0025–0.0034			Lakes, ponds, rivers	Non-invasive genetic CMR
Kruuk et al. [5]			0.5–0.7	Coastal habitat	Census of otter holts
Lanszki et al. [10]			0.17	Rivers, backwater	Non-invasive microsatellite genotyping (MNA)
Lanszki et al. [27]		0.018–0.046	0.35–1.2	Fish ponds	Non-invasive microsatellite genotyping (MNA)
Prigioni et al. [59]			0.18–0.2	Rivers	Non-invasive microsatellite genotyping (AC)
Ruiz-Olmo et al. [60]		0.015–0.063	0.07–0.26	Rivers	Direct census
Sidorovich [30]			0.02–0.4	Rivers, backwater	Direct census, tracking of footprints

CMR—capture-mark-recapture analyses

MNA—minimum number alive (no estimation only number of genotypes)

AC—accumulation curve, e.g. [63]

For a comparison, we included results of this study (bold).

### Marking Behaviour and Impacts on CMR Analyses

We showed that although the number of markings did not significantly vary between sexes, jelly samples (with higher success rates) were more frequently defecated by males and placed exposed on previously used marking sites with several old/fresh scats. Hence, male-biases could be introduced by preferring those kind of samples or these „hot spots”that are usually larger and more prominent, thus easier to find (e.g. markings sites under bridges). Therefore, we agree with Bonesi et al. [16] that non-invasive genetic sampling on otters has to account for their marking behaviour to gain information about sex ratios. Our results indicate that it could be crucial to not drop too many low quality samples, but to invest in replications increasing the overall genotyping success and the numbers of females successfully genotyped, and to include all kinds of marking sites in a study design, also less frequently used sites, to minimise the risk of collecting only a fraction of a population.

We found an even sex ratio in 2008 and more females in 2007/10/11/12. Years 2011/12 even showed non-overlapping 95% confidence intervals (CI) between the number of estimated females and males (Table 2) and thus a female-bias. Although we found slightly more males in 2006, the CIs of males and females broadly overlapped, indicating no male-bias. The true sex ratio of otter populations is so far unknown, but a female-bias is to be expected because of an almost equal ratio for new born (♂/♀ = 1.125) or three months old cups (♂/♀ = 1.09) [30] but lower female mortalities [64]. A female-biased sex ratio was also observed by Kruuk [1] (♂/♀ = 0.83). However, most studies employing non-invasive genetic sampling [15–17, 25–28] found more males both in number of samples and individuals. Therefore, Bonesi et al. [16] questioned the usefulness of non-invasive sampling to estimate population size and sex ratios of otters. Our balanced or female-biased sex ratios might be explained by persistent repetitions of lower quality samples, dropping only samples with no chances to gain a complete multilocus genotype, and by including all kind of marking sites in the sampling.

The fact that jelly samples are more often defecated by males also indicates that especially jelly samples have a special role either in sexual communication or for another sex-dependent function, such as social status as found for river otters [65]. Whereas Kruuk [1], Kruuk [66] postulated that spraints have probably no function in territory defence or sexual communication but in resource partitioning, a function in sexual communication was also postulated by Remonti et al. [67] and Kean et al. [33]. The latter demonstrated that volatile compounds from anal gland secretions differed in age and for adults also in sex and with reproductive status.

### Behavioural Response of Sampled Otters

Compared to the capture rates, we observed higher recapture rates in almost all sampling years—except for 2010, when both rates were comparably high. This could be due to a changed sampling protocol in 2010: larger faeces were first sampled with a cotton swab for genetic analyses and then entirely taken for hormone analyses. In all other years faecal samples were not removed. As otters reuse their marking sites for many years and also daily [1], higher recapture rates could be collector-induced if they searched more intensely on known marking sites or if they found more samples after a settling-in period (e.g. first 1–2 days). However, 71.1% of the individuals either never reused marking sites (45.9%) or reused one marking site at maximum twice within the five sampling days (25.2%). We also found no difference in the sampling patterns (e.g. settling-in period) between expert collectors and students. Another possibility is that already collected otters reacted on the frequent treatment of their spraints with an increased marking intensity. Such a behavioural response is known as “trap-happiness”. It is known that otters use spraints for intraspecific communication [1, 66] and so it could well be that they will notice if somebody handled and thus altered their markings. This could put them on the alert resulting in a higher marking intensity. Such behaviour was also found by Brzezinski and Romanowski [31], who conducted an experimental approach and found higher sprainting intensity on sites where spraints were previously removed. Removing spraints in 2010 may have disturbed the intraspecific communication such that also unsampled individuals increased their marking intensity or at least used marking sites that were seemingly free of any usage because of previous faecal removing. This is reasonable as the same marking site was used by up to six different individuals within five sampling days [64]. Regardless of whether the behavioural effect is collector- or otter-induced, it is important to account for this when estimating population size of otters (i.e. by including M_b_), otherwise the results can be severely biased.

According to summed AIC_c_ weights for each effect (constant, heterogeneity, behavioural, time) following Burnham and Anderson [57], a behavioural effect (incorporated in model M_b_, M_bh_, M_tb_, M_tb_constrained_) was the most important when averaging over years (0.4) followed by time (0.36), heterogeneity (0.32), and constant (0.22).

### Otter Monitoring by Spraint Densities

It has been argued that spraint density can be used as an index of abundance for comparison of populations in time or in space [8] and has been applied in several studies (see [68] for a review). A non-invasive genetic study even found a significant positive relation between spraint density and number of genotypes per area [10]. In our study this relationship also was close to significance. However, when relating the spraint density with the number of estimated individuals there was no correlation. Even when comparing only the four sampling years (2006, 2010–2012) where we always sampled end of March, there was no relationship between number of individuals and samples (R^2^ = 0.02, df = 2, p = 0.87). This can be explained by the removal of ghost individuals, which was not the case in the study by Lanszki et al. [10], who used the number of sampled genotypes instead of a population size estimate. It is natural that the more samples one collects in an area or period, the more ghost individuals will be in the dataset and thus the more genotypes one will have. Hence, in line with other authors [13, 14], we caution against the extrapolation of otter spraint densities to relative abundances.

Furthermore, although we did not change our sampling design or the way of sampling, the number of collected mink scats increased tremendously in years 2010–2012 compared to 2006–2008 and was about two to six-fold higher. This increase was accompanied by an increased mink density from 2010 on as shown by the numbers of harvested minks with MPT_2010_ = 0.091. For comparison, a saturated mink population in ca. 120 ha of the river Thames amounted to MPT = 0.04 using live-traps and including recaptures [69]. This implies that contrary to most studies stating that high otter densities are likely to entail a decline in mink densities [70–72], the mink proliferated quite well in our study area despite high otter densities. Similarly, Harrington et al. [73] found that mink abundances remained relatively high while otter densities raised.

Bonesi and Macdonald [74] stated that mink may persist in the presence of otters when terrestrial prey is abundant. The Upper Lusatian pond landscape is known for a high diversity in amphibians, reptiles, water birds, and small mammals [37]. However, most of the mink scats were collected because they contained fish remains, making them more similar to otter spraints. If minks coexist with otters, Bueno [75] found that minks prey on smaller fishes than otters, which might well be so for our study area. Beside mink scats containing fish remains, we also unintentionally collected mink scats that looked like otter jelly samples. Dunstone [76] already pointed out that mink can produce a jelly-like secretion. The mink samples were not only collected by students but also by expert collectors. The same difficulty was already noted by Harrington et al. [77]. In their study not a single supposed mink sample collected by experts was of mink origin; rather they belonged to pine martens (47%), foxes (41%), otters (6%), polecats (3%), or stoats (3%). In our study, fresh mink samples were found on typical otter marking sites, sometimes next to fresh otter samples from the same night. This implies that otter monitoring solely relying on otter spraints without genetically determining the species run the risk of overestimating abundance or occupancy if minks are present.

An extrapolation from spraint densities to otter densities is even more precarious to use given that a) number of samples vary seasonally [78], b) sampling rate (collector-induced) or marking intensity (otter-induced) can increase during several-day sampling periods (see discussion above), and c) one marking site is used by up to six individuals [64].

## Conclusion

Faeces are a valuable source to gain information about population sizes and sex ratios via the use of genetic mark-recapture when potential error sources are carefully addressed and the marking behaviour of the target species is taken into account. We illustrated how sex differences in the marking behaviour can influence non-invasive genetic CMR, because high DNA quality jelly samples were more often defecated by males than by females and placed exposed on frequently used marking sites that are easier to find for collectors. Hence, it is crucial to not only concentrate on sampling jelly samples or on prominent marking sites. Furthermore, we recommend investing in high genotyping success rates by sufficient numbers of repetitions to ensure unbiased sex ratios and decreased genotyping error rates. Because of either collector-induced varying sampling intensity or a behavioural response of otters on spraint handling and removing, researchers should employ models that can account for a behavioural effect to receive unbiased estimates. Even when using high quality samples, researchers should use CMR models that incorporate genotyping errors to avoid overestimates, since it is difficult to completely exclude genotyping errors [23]. Our study further shows that faecal densities are not a reliable index for otter abundances because of variability in marking behaviour and because of the risk of confusion with mink faeces even by experts. Similar problems may exist for other elusive species. Therefore, we strongly recommend testing the reliability of faecal densities as index of abundance with genetic CMR methods before using them for monitoring elusive species.

## Supporting Information

S1 Supporting Information Details on DNA extraction, amplification, and microsatellite genotyping. (DOCX)

S1 Table Supplementary table showing genotypes and spraint characteristics of all successfully genotyped otter samples. (PDF)

## References

[1] Kruuk H. Otters—ecology, behaviour and conservation Oxford, New York: Oxford University Press; 2006.

[2] Ruiz-Olmo J, Loy A, Cianfrani C, Yoxon P, Yoxon G, de Silva PK, et al. *Lutra lutra*: www.iucnredlist.org; 2008 [cited 2014 08 April 2014]. Version 2013.2:[

[3] Kruuk H, Moorhouse A. The spatial organization of otters (*Lutra lutra*) in Shetland. J Zool. 1991;224:41–57.

[4] Ruiz-Olmo J, Saavedra D, Jiménez J. Testing the surveys and visual and track censuses of Eurasian otters (*Lutra lutra*). J Zool. 2001;253(3):359–69.

[5] Kruuk H, Moorhouse A, Conroy JWH, Durbin L, Frears S. An estimate of numbers and habitat preferences of otters *Lutra lutra* in Shetland, UK. Biol Conserv. 1989;49(4):241–54.

[6] Garcia-Diaz P, Arevalo V, Lizana M. Comparison of track and direct observation estimations for assessing abundance of the Eurasian otter, *Lutra lutra* . Folia Zoologica. 2011;60(1):37–42.

[7] Sulkava R. Snow tracking: a relevant method for estimating otter *Lutra lutra* populations. Wildlife Biol. 2007;13(2):208–18.

[8] Mason CF, Macdonald SM. The use of spraints for surveying otter *Lutra lutra* populations: An evaluation. Biol Conserv. 1987;41(3):167–77.

[9] Balestrieri A, Remonti L, Prigioni C. Detectability of the Eurasian otter by standard surveys: an approach using marking intensity to estimate false negative rates. Naturwissenschaften. 2011;98(1):23–31. 10.1007/s00114-010-0737-0 21080153

[10] Lanszki J, Hidas A, Szentes K, Revay T, Lehoczky I, Weiss S. Relative spraint density and genetic structure of otter (*Lutra lutra*) along the Drava River in Hungary. Mamm Biol. 2008;73(1):40–7.

[11] Guter A, Dolev A, Saitz D, Kronfeld-Schor N. Using videotaping to validate the use of spraints as an index of Eurasian otter (*Lutra lutra*) activity. Ecol Indic. 2008;8(5):462–5. 10.3171/SPI/2008/8/5/462 18447693

[12] Calzada J, Delibes-Mateos M, Clavero M, Delibes M. If drink coffee at the coffee-shop is the answer, what is the question? Some comments on the use of the sprainting index to monitor otters. Ecol Indic. 2010;10(2):560–1.

[13] Chanin P. Monitoring the otter *Lutra lutra* Petersborough, UK: English Nature, 2003.

[14] Kruuk H, Conroy JWH, Glimmerveen U, Ouwerkerk EJ. The use of spraints to survey populations of otters *Lutra lutra* . Biol Conserv. 1986;35(2):187–94.

[15] Hajkova P, Zemanova B, Roche K, Hajek B. An evaluation of field and noninvasive genetic methods for estimating Eurasian otter population size. Conserv Genet. 2009;10(6):1667–81.

[16] Bonesi L, Hale M, Macdonald DW. Lessons from the use of non-invasive genetic sampling as a way to estimate Eurasian otter population size and sex ratio. Acta Theriol. 2013;58(2):157–68.

[17] Arrendal J, Vila C, Bjorklund M. Reliability of noninvasive genetic census of otters compared to field censuses. Conserv Genet. 2007;8(5):1097–107.

[18] Höss M, Kohn M, Paabo S, Knauer F, Schroder W. Excrement analysis by PCR. Nature. 1992;359(6392):199–. 1528260 10.1038/359199a0

[19] Taberlet P, Bouvet J. Bear conservation genetics. Nature. 1992;358(6i383):197–. 1630487 10.1038/358197a0

[20] Marucco F, Boitani L, Pletscher DH, Schwartz MK. Bridging the gaps between non-invasive genetic sampling and population parameter estimation. Eur J Wildl Res. 2011;57(1):1–13.

[21] Lukacs PM, Burnham KP. Review of capture-recapture methods applicable to noninvasive genetic sampling. Mol Ecol. 2005;14(13):3909–19. 16262847 10.1111/j.1365-294X.2005.02717.x

[22] Pompanon F, Bonin A, Bellemain E, Taberlet P. Genotyping errors: Causes, consequences and solutions. Nat Rev Genet. 2005;6(11):847–59. 16304600 10.1038/nrg1707

[23] Lampa S, Henle K, Klenke R, Hoehn M, Gruber B. How to overcome genotyping errors in non-invasive genetic mark-recapture population size estimation—A review of available methods illustrated by a case study. J Wildl Manage. 2013;77(8):1490–511.

[24] Creel S, Spong G, Sands JL, Rotella J, Zeigle J, Joe L, et al Population size estimation in Yellowstone wolves with error-prone noninvasive microsatellite genotypes. Mol Ecol. 2003;12(7):2003–9. 12803649 10.1046/j.1365-294x.2003.01868.x

[25] Kalz B, Jewgenow K, Fickel J. Structure of an otter (*Lutra lutra*) population in Germany—results of DNA and hormone analyses from faecal samples. Mamm Biol. 2006;71(6):321–35.

[26] Janssens X, Fontaine MC, Michaux JR, Libois R, Kermabon Jd, Defourny P, et al Genetic pattern of the recent recovery of European otters in southern France. Ecography. 2008;31(2):176–86.

[27] Lanszki J, Hidas A, Szentes K, Revay T, Lehoczky I, Jeney Z, et al Genetic structure of otter (*Lutra lutra*) populations from two fishpond systems in Hungary. Mamm Biol. 2010;75(5):447–50.

[28] Dallas JF, Coxon KE, Sykes T, Chanin PRF, Marshall F, Carss DN, et al Similar estimates of population genetic composition and sex ratio derived from carcasses and faeces of Eurasian otter *Lutra lutra* . Mol Ecol. 2003;12:275–82. 12492895 10.1046/j.1365-294x.2003.01712.x

[29] Ansorge H, Schipke R, Zinke O. Population structure of the otter, *Lutra lutra*. Parameters and model for a Central European region. Mamm Biol. 1997;62:142–51.

[30] Sidorovich VE. Structure, reproductive status and dynamics of the otter population in Byelorussia. Acta Theriol. 1991;36(1–2):153–61.

[31] Brzezinski M, Romanowski J. Experiments on sprainting activity of otters *(Lutra lutra*) in the Bieszczady Mountains, southeastern Poland. Mammalia. 2006;70(1–2):58–63.

[32] Trowbridge BJ. Olfactory communication in the European otter (*Lutra lutra*) Aberdeen (UK): University of Aberdeen; 1983.

[33] Kean EF, Muller CT, Chadwick EA. Otter Scent Signals Age, Sex, and Reproductive Status. Chem Senses. 2011;36(6):555–+. 10.1093/chemse/bjr025 21444931

[34] Lampa S, Gruber B, Henle K, Hoehn M. An optimisation approach to increase DNA amplification success of otter faeces. Conserv Genet. 2008;9(1):201–10. 10.1186/gb-2008-9-1-201 18226181 PMC2395230

[35] Hajkova P, Zemanova B, Bryja J, Hajek B, Roche K, Tkadlec E, et al Factors affecting success of PCR amplification of microsatellite loci from otter faeces. Mol Ecol Notes. 2006;6(2):559–62.

[36] Myšiak J, Schwerdtner-Máñez K, Ring I. Comparative analysis of the conflicts between carp pond farming and the protection of otters (*Lutra lutra*) in Upper Lusatia and South Bohemia In: Klenke RA, Ring I, Kranz A, Jepsen N, Rauschmayer F, Henle K, editors. Human—Wildlife Conflicts in Europe Fisheries and Fish-eating Vertebrates as a Model Case. Evironmental Science and Engineering Berlin, Heidelberg: Springer; 2013 p. 141–62.

[37] Böhnert W, Buchwald RG, Reichoff L. Analyse und Bewertung des Landschaftsraumes—Fauna Böhnert W, Buchwald RG, Reichoff L, editors. Mücka: Biosphärenreservat Oberlausitzer Heide- und Teichlandschaft; 1996.

[38] Klenke R. Ergebnisse der Erfassung von Fischotternachweisen von 1993 bis 1995 In: Geologie SLfUu, editor. Artenschutzprogramm—Fischotter in Sachsen. Radebeul: Materialien zu Naturschutz und Landschaftspflege; 1996 p. 12–7.

[39] Ansorge H. Zur Situation des eurasischen Fischotters *Lutra lutra* Linné, 1758 im Raum Oberlausitz-Sachsen. Säugetierkundliche Informationen. 1994;3(18):617–22.

[40] Hauer S, Ansorge H, Zinke O. Reproductive performance of otters *Lutra lutra* (Linnaeus, 1758) in Eastern Germany: low reproduction in a long-term strategy. Biol J Linnean Soc. 2002;77(3):329–40.

[41] Dallas JF, Piertney SB. Microsatellite primers for the Eurasian otter. Mol Ecol. 1998;7:1247–63. 9734080

[42] Dallas JF, Marshall F, Piertney SB, Bacon PJ, Racey PA. Spatially restricted gene flow and reduced microsatellite polymorphism in the Eurasian otter *Lutra lutra* in Britain. Conserv Genet. 2002;3:15–29.

[43] Dallas JF, Carss DN, Marshall F, Koepfli K-P, Kruuk H, Piertney SB, et al Sex identification of the Eurasian otter *Lutra lutra* by PCR typing of spraints. Conserv Genet. 2000;1:181–3.

[44] Hedmark E, Flagstad O, Segerstrom P, Persson J, Landa A, Ellegren H. DNA-based individual and sex identification from wolverine (*Gulo gulo*) faeces and urine. Conserv Genet. 2004;5(3):405–10.

[45] Koelewijn HP, Perez-Haro M, Jansman HAH, Boerwinkel MC, Bovenschen J, Lammertsma DR, et al The reintroduction of the Eurasian otter (*Lutra lutra*) into the Netherlands: hidden life revealed by noninvasive genetic monitoring. Conserv Genet. 2010;11(2):601–14.

[46] McKelvey KS, Schwartz MK. DROPOUT: a program to identify problem loci and samples for noninvasive genetic samples in a capture-mark-recapture framework. Mol Ecol Notes. 2005;5(3):716–8.

[47] Broquet T, Petit E. Quantifying genotyping errors in noninvasive population genetics. Mol Ecol. 2004;13(11):3601–8. 15488016 10.1111/j.1365-294X.2004.02352.x

[48] Valière N. GIMLET: a computer program for analysing genetic individual identification data. Mol Ecol Notes. 2002;2(3):377–9.

[49] Holm S. A simple sequentially rejective multiple test procedure. Scand J Stat. 1979;6(2):65–70.

[50] Hothorn T, Hornik K. exactRankTests: Exact distribution for rank and permutation tests. R package version 0.8–27, Available: http://cran.r-project.org/web/packages/exactRankTests/index.html. Accessed 26th Nov 2013.

[51] R Core Team. R: A language and environment for statistical computing R Foundation for Statistical Computing Vienna, Austria: Available: http://www.R-project.org; 2013.

[52] Pollock KH, Nichols JD, Brownie C, Hines JE. Statistical inference for capture-recapture experiments. Wildl Monogr. 1990;(107):1–97.

[53] Lukacs PM, Burnham KP. Estimating population size from DNA-based closed capture-recapture data incorporating genotyping error. J Wildl Manage. 2005;69(1):396–403.

[54] White GC, Burnham KP. Program MARK: survival estimation from populations of marked animals. Bird Stud. 1999;46:120–39.

[55] Hurvich CM, Tsai CL. Regression and time-series model selection in small samples. Biometrika. 1989;76(2):297–307.

[56] Sugiura N. Further analysis of the data by Akaike's information criterion and the finite corrections. Communications in Statistics—Theory and Methods. 1978;(A 7):13–26.

[57] Burnham KP, Anderson DR. Model selection and multimodel inference: A practical information-theoretic approach 2nd ed. ed. New York: Springer-Verlag; 2002.

[58] Hung CM, Li SH, Lee LL. Faecal DNA typing to determine the abundance and spatial organisation of otters (*Lutra lutra*) along two stream systems in Kinmen. Anim Conserv. 2004;7:301–11.

[59] Prigioni C, Remonti L, Balestrieri A, Sgrosso S, Priore G, Mucci N, et al Estimation of European otter (*Lutra lutra*) population size by feacal DNA typing in Southern Italy. J Mammal. 2006;87(5):855–8.

[60] Ruiz-Olmo J, Batet A, Manas F, Martinez-Vidal R. Factors affecting otter (*Lutra lutra*) abundance and breeding success in freshwater habitats of the northeastern Iberian Peninsula. Eur J Wildl Res. 2011;57(4):827–42.

[61] Erlinge S. Territoriality of the Otter *Lutra lutra* L. Oikos. 1968;19(1):81–98.

[62] Miller CR, Joyce P, Waits LP. A new method for estimating the size of small populations from genetic mark-recapture data. Mol Ecol. 2005;14(7):1991–2005. 15910321 10.1111/j.1365-294X.2005.02577.x

[63] Kohn MH, York EC, Kamradt DA, Haught G, Sauvajot RM, Wayne RK. Estimating population size by genotyping faeces. Royal Society. 1999;266:657–63. 10331287 10.1098/rspb.1999.0686 PMC1689828

[64] Lampa S, Mihoub JB, Gruber B, Klenke R, Henle K. Non-invasive genetic mark-recapture as a means to study population dynamic and spatial use of Eurasian otters (*Lutra lutra*) in a fish pond landscape. PLoS One. subm. 10.1371/journal.pone.0125684 PMC4431875 25973884

[65] Rostain RR, Ben-David M, Groves P, Randall JA. Why do river otters scent-mark? An experimental test of several hypotheses. Animal Behaviour. 2004;68:703–11.

[66] Kruuk H. Scent marking by otters (*Lutra lutra*)—signaling the use of resources. Behav Ecol. 1992;3(2):133–40.

[67] Remonti L, Balestrieri A, Smiroldo G, Prigioni C. Scent marking of key food sources in the Eurasian otter. Ann Zool Fenn. 2011;48(5):287–94.

[68] Reuther C, Dolch D, Green R, Jahrl J, Jefferies DJ, Krekemeyer A, et al Surveying and monitoring distribution and population trends of the Eurasian otter (*Lutra lutra*). Habitat. 2000;12:1–148.

[69] Yamaguchi N, MacDonald DW. The Burden of Co-Occupancy: Intraspecific Resource Competition and Spacing Patterns in American Mink, *Mustela vison* . J Mammal. 2003;84(4):1341–55.

[70] Bonesi L, Macdonald DW. Impact of released Eurasian otters on a population of American mink: a test using an experimental approach. Oikos. 2004;106(1):9–18.

[71] Bonesi L, Strachan R, Macdonald DW. Why are there fewer signs of mink in England? Considering multiple hypotheses. Biol Conserv. 2006;130(2):268–77.

[72] McDonald RA, O'Hara K, Morrish DJ. Decline of invasive alien mink (*Mustela vison*) is concurrent with recovery of native otters (*Lutra lutra*). Divers Distrib. 2007;13(1):92–8.

[73] Harrington LA, Harrington AL, Yamaguchi N, Thom MD, Ferreras P, Windham TR, et al The impact of native competitors on an alien invasive: temporal niche shifts to avoid interspecific aggression? Ecology. 2009;90(5):1207–16. 19537542 10.1890/08-0302.1

[74] Bonesi L, Macdonald DW. Differential habitat use promotes sustainable coexistence between the specialist otter and the generalist mink. Oikos. 2004;106(3):509–19.

[75] Bueno F. Competition between American mink *Mustela vison* and otter L*utra lutra* during winter. Acta Theriol. 1996;41(2):149–54.

[76] Dunstone N. The mink London, UK: T. & A.D. Poyser Ltd; 1993. 232 p.

[77] Harrington LA, Harrington AL, Hughes J, Stirling D, Macdonald DW. The accuracy of scat identification in distribution surveys: American mink, *Neovison vison*, in the northern highlands of Scotland. Eur J Wildl Res. 2010;56(3):377–84.

[78] Macdonald SM, Mason CF. Seasonal marking in an otter population. Acta Theriol. 1987;32(21–31):449–62.

