# Supplementary material for: Non-Invasive Genetic Mark-Recapture as a Means to Study Population Sizes and Marking Behaviour of the Elusive Eurasian Otter (Lutra lutra)
Source: PLoS One. 2015 May 14;10(5):e0125684. doi: 10.1371/journal.pone.0125684 (PMC4431875; doi:10.1371/journal.pone.0125684)
Supplement: S1 Supporting Information — (DOCX) [file pone.0125684.s001.docx]

**S1 Supporting Information**

**Details on DNA Extraction, Amplification and Microsatellite Genotyping**

DNA was extracted from all samples employing the QIAamp^®^ DNA Stool Mini Kit (Qiagen), starting with either adding warm ASL buffer (70°C) to samples (year 2006) or warming up samples to 70°C. All samples were then vortexed and incubated for 2 min at room temperature before proceeding with step four in the manufacturer’s protocol. DNA extracts were afterwards stored at –20°C.

All forward primers were fluorescence labelled with the dyes FAM, HEX (Eurofins MWG Operon, Ebersberg, Germany), or NED (Applied Biosystems, Darmstadt, Germany). To enhance comparability of DNA fragments, the pigtail ‘GTTGCTT’ was added to the 5´-end of reverse primers to generate a poly(A) tail at the 3´-end. Samples of the year 2006 were amplified following a variation of the multiplex pre-amplification ([1](#_ENREF_1), [2](#_ENREF_2)), in which two consecutive polymerase chain reactions (PCR) are carried out for each primer set ([see 3](#_ENREF_3)), to increase genotyping success rates and to lower genotyping error rates. For samples of 2007–2012, we were able to gain the same success with only one single PCR using a more sensitive polymerase enzyme with high-fidelity and hot-start technique (AmpliTaq Gold^®^ 360 DNA Polymerase, Applied Biosystems) under the following conditions: 25 µl reaction volumes consisted of 3 µl DNA extract, 12.5 µl AmpliTaq Gold^®^ 360 Master Mix, 0.6 µM of each primer, and HPLC-water to the total volume. The hot-start Taq polymerase required an initial denaturation of 95°C for 10 min, followed by 45 cycles of 95°C for 30 sec, 58°C (M1, M2) or 56°C (M3) for 1 min, and 72°C for 30 sec, ending with a final extension at 72°C for 7 min. PCR products were separated and visualised in an ABI PRISM^®^ 3100 Genetic Analyser and analysed using ABI PRISM^®^ GeneMapper™ Software V.3.7 (Applied Biosystems, Darmstadt, Germany).

For the additional repetitions after the fifth amplification step of the applied screening approach, we partly employed the pre-amplification approach described above and/or used a G/C-Enhancer buffer (included in the AmpliTaq Gold^®^ 360 Master Mix) to increase success rates and to lower genotyping error rates.

**References**

1. Piggott MP, Bellemain E, Taberlet P, Taylor AC. A multiplex pre-amplification method that significantly improves microsatellite amplification and error rates for faecal DNA in limiting conditions. Conserv Genet. 2004;5(3):417-20.

2. Bellemain E, Taberlet P. Improved noninvasive genotyping method: application to brown bear (*Ursus arctos*) faeces. Mol Ecol Notes. 2004;4(3):519-22.

3. Lampa S, Gruber B, Henle K, Hoehn M. An optimisation approach to increase DNA amplification success of otter faeces. Conserv Genet. 2008;9(1):201-10.
