# Supplementary material for: Non-Invasive Genetic Mark-Recapture as a Means to Study Population Sizes and Marking Behaviour of the Elusive Eurasian Otter (Lutra lutra)
Source: PLoS One. 2015 May 14;10(5):e0125684. doi: 10.1371/journal.pone.0125684 (PMC4431875; doi:10.1371/journal.pone.0125684)
Supplement: S1 Table — (PDF) [file pone.0125684.s002.pdf]

S1 Table: Supplementary table showing genotypes and spraint characteristics of all successfully genotyped otter samples

Sample ID	Otter ID	Collection date	Size of spraint	Sliminess	Exposure level	Sample numbers on site	Lut435		Lut604		Lut701		Lut457		Lut615		Lut733		Lut914		Sex
**A003**	2	27.03.2006	small	mucous spraint	actively exposed	>4	129	129	135	137	211	211	135	135	140	142	172	196	150	150	M
**A007**	8	27.03.2006	large	jelly	passively exposed	3-4	145	145	139	141	211	211	135	141	140	142	176	196	150	150	M
**A009**	8	27.03.2006	large	jelly	passively exposed	>4	145	145	139	141	211	211	135	141	140	142	176	196	150	150	M
**A011**	6	27.03.2006	large	jelly	passively exposed	3-4	129	145	135	139	211	211	141	151	140	142	172	176	150	150	F
**A013**	7	27.03.2006	large	jelly	actively exposed	>4	129	147	135	139	211	211	135	135	140	148	176	180	146	150	M
**A028**	16	27.03.2006	large	spraint	non-exposed	3-4	129	145	137	141	211	211	135	151	140	142	172	176	146	146	F
**A033**	14	27.03.2006	small	jelly	non-exposed	1-2	129	145	137	139	211	211	135	151	140	142	176	180	146	150	M
**A085**	20	27.03.2006	medium	mucous spraint	non-exposed	3-4	129	145	135	137	211	211	135	135	142	144	180	180	146	150	M
**A087**	20	27.03.2006	small	spraint	actively exposed	3-4	129	145	135	137	211	211	135	135	142	144	180	180	146	150	M
**A122**	10	27.03.2006	small	spraint	non-exposed	1-2	129	145	135	139	203	211	135	141	140	142	180	196	150	150	M
**A125**	13	27.03.2006	medium	spraint	non-exposed	1-2	129	145	139	141	211	211	135	141	140	142	172	180	150	150	F
**A127**	10	27.03.2006	medium	mucous spraint	actively exposed	3-4	129	145	135	139	203	211	135	141	140	142	180	196	150	150	M
**A130**	11	27.03.2006	medium	mucous spraint	passively exposed	3-4	129	145	135	141	203	211	141	141	140	142	176	180	150	150	M
**A131**	11	27.03.2006	medium	mucous spraint	passively exposed	3-4	129	145	135	141	203	211	141	141	140	142	176	180	150	150	M
**A133**	10	27.03.2006	medium	mucous spraint	passively exposed	>4	129	145	135	139	203	211	135	141	140	142	180	196	150	150	M
**A137**	11	27.03.2006	small	jelly	non-exposed	1-2	129	145	135	141	203	211	141	141	140	142	176	180	150	150	M
**A138**	12	27.03.2006	small	mucous spraint	actively exposed	1-2	145	145	137	139	203	211	135	141	140	142	176	180	150	150	F
**A139**	13	27.03.2006	small	jelly	passively exposed	3-4	129	145	139	141	211	211	135	141	140	142	172	180	150	150	F
**B061**	14	28.03.2006	large	mucous spraint	passively exposed	>4	129	145	137	139	211	211	135	151	140	142	176	180	146	150	M
**B062**	16	28.03.2006	small	mucous spraint	non-exposed	3-4	129	145	137	141	211	211	135	151	140	142	172	176	146	146	F
**B069**	16	28.03.2006	large	mucous spraint	non-exposed	3-4	129	145	137	141	211	211	135	151	140	142	172	176	146	146	F
**B070**	16	28.03.2006	large	spraint	non-exposed	3-4	129	145	137	141	211	211	135	151	140	142	172	176	146	146	F
**B076**	17	28.03.2006	large	mucous spraint	non-exposed	>4	145	145	137	139	211	211	135	135	140	142	176	180	146	150	F
**B078**	17	28.03.2006	large	mucous spraint	non-exposed	>4	145	145	137	139	211	211	135	135	140	142	176	180	146	150	F
**B079**	16	28.03.2006	large	spraint	non-exposed	>4	129	145	137	141	211	211	135	151	140	142	172	176	146	146	F
**B080**	14	28.03.2006	large	jelly	passively exposed	1-2	129	145	137	139	211	211	135	151	140	142	176	180	146	150	M
**B081**	20	28.03.2006	small	mucous spraint	passively exposed	>4	129	145	135	137	211	211	135	135	142	144	180	180	146	150	M
**B082**	20	28.03.2006	small	mucous spraint	non-exposed	>4	129	145	135	137	211	211	135	135	142	144	180	180	146	150	M
**B088**	20	28.03.2006	small	mucous spraint	non-exposed	1-2	129	145	135	137	211	211	135	135	142	144	180	180	146	150	M
**B098**	20	28.03.2006	medium	spraint	non-exposed	1-2	129	145	135	137	211	211	135	135	142	144	180	180	146	150	M
**B103**	10	28.03.2006	large	jelly	non-exposed	3-4	129	145	135	139	203	211	135	141	140	142	180	196	150	150	M
**B106**	10	28.03.2006	medium	mucous spraint	passively exposed	3-4	129	145	135	139	203	211	135	141	140	142	180	196	150	150	M
**B109**	10	28.03.2006	medium	mucous spraint	non-exposed	>4	129	145	135	139	203	211	135	141	140	142	180	196	150	150	M
**B111**	13	28.03.2006	small	mucous spraint	non-exposed	1-2	129	145	139	141	211	211	135	141	140	142	172	180	150	150	F
**B112**	10	28.03.2006	medium	mucous spraint	non-exposed	1-2	129	145	135	139	203	211	135	141	140	142	180	196	150	150	M
**B113**	11	28.03.2006	small	mucous spraint	passively exposed	>4	129	145	135	141	203	211	141	141	140	142	176	180	150	150	M
**B142**	4	28.03.2006	medium	spraint	passively exposed	1-2	129	145	135	137	211	211	135	135	140	142	196	196	146	150	F
**B144**	1	28.03.2006	medium	mucous spraint	actively exposed	>4	129	129	135	137	211	211	135	135	140	142	172	180	150	150	F

**B147**	1	28.03.2006	small	spraint	actively exposed	3-4	129	129	135	137	211	211	135	135	140	142	172	180	150	150	F
**B148**	1	28.03.2006	medium	mucous spraint	actively exposed	3-4	129	129	135	137	211	211	135	135	140	142	172	180	150	150	F
**B151**	1	28.03.2006	medium	spraint	passively exposed	3-4	129	129	135	137	211	211	135	135	140	142	172	180	150	150	F
**B153**	6	28.03.2006	large	mucous spraint	passively exposed	>4	129	145	135	139	211	211	141	151	140	142	172	176	150	150	F
**B158**	1	28.03.2006	medium	mucous spraint	actively exposed	>4	129	129	135	137	211	211	135	135	140	142	172	180	150	150	F
**C062**	13	29.03.2006	small	spraint	non-exposed	1-2	129	145	139	141	211	211	135	141	140	142	172	180	150	150	F
**C063**	13	29.03.2006	small	mucous spraint	non-exposed	1-2	129	145	139	141	211	211	135	141	140	142	172	180	150	150	F
**C064**	10	29.03.2006	large	mucous spraint	passively exposed	3-4	129	145	135	139	203	211	135	141	140	142	180	196	150	150	M
**C067**	10	29.03.2006	small	mucous spraint	passively exposed	3-4	129	145	135	139	203	211	135	141	140	142	180	196	150	150	M
**C068**	10	29.03.2006	small	jelly	non-exposed	>4	129	145	135	139	203	211	135	141	140	142	180	196	150	150	M
**C069**	10	29.03.2006	medium	jelly	non-exposed	>4	129	145	135	139	203	211	135	141	140	142	180	196	150	150	M
**C070**	11	29.03.2006	medium	mucous spraint	non-exposed	1-2	129	145	135	141	203	211	141	141	140	142	176	180	150	150	M
**C084**	16	29.03.2006	large	spraint	non-exposed	1-2	129	145	137	141	211	211	135	151	140	142	172	176	146	146	F
**C086**	18	29.03.2006	small	mucous spraint	non-exposed	>4	129	129	135	141	211	211	135	135	142	144	172	180	146	150	F
**C089**	17	29.03.2006	large	mucous spraint	passively exposed	3-4	145	145	137	139	211	211	135	135	140	142	176	180	146	150	F
**C090**	16	29.03.2006	large	mucous spraint	passively exposed	3-4	129	145	137	141	211	211	135	151	140	142	172	176	146	146	F
**C095**	17	29.03.2006	large	mucous spraint	passively exposed	3-4	145	145	137	139	211	211	135	135	140	142	176	180	146	150	F
**C096**	16	29.03.2006	large	mucous spraint	passively exposed	3-4	129	145	137	141	211	211	135	151	140	142	172	176	146	146	F
**C097**	17	29.03.2006	medium	mucous spraint	passively exposed	3-4	145	145	137	139	211	211	135	135	140	142	176	180	146	150	F
**C104**	2	29.03.2006	medium	mucous spraint	actively exposed	>4	129	129	135	137	211	211	135	135	140	142	172	196	150	150	M
**C105**	3	29.03.2006	large	jelly	actively exposed	>4	129	145	135	137	211	211	135	135	140	142	172	196	150	150	M
**C109**	6	29.03.2006	medium	jelly	actively exposed	1-2	129	145	135	139	211	211	141	151	140	142	172	176	150	150	F
**C110**	6	29.03.2006	medium	jelly	actively exposed	1-2	129	145	135	139	211	211	141	151	140	142	172	176	150	150	F
**C111**	8	29.03.2006	large	jelly	actively exposed	3-4	145	145	139	141	211	211	135	141	140	142	176	196	150	150	M
**C112**	7	29.03.2006	medium	mucous spraint	actively exposed	>4	129	147	135	139	211	211	135	135	140	148	176	180	146	150	M
**C117**	7	29.03.2006	medium	jelly	actively exposed	>4	129	147	135	139	211	211	135	135	140	148	176	180	146	150	M
**C119**	6	29.03.2006	large	jelly	actively exposed	>4	129	145	135	139	211	211	141	151	140	142	172	176	150	150	F
**C120**	2	29.03.2006	medium	mucous spraint	actively exposed	>4	129	129	135	137	211	211	135	135	140	142	172	196	150	150	M
**C150**	19	29.03.2006	medium	mucous spraint	non-exposed	1-2	129	129	135	141	207	211	135	141	142	144	180	180	146	150	F
**C152**	21	29.03.2006	medium	mucous spraint	actively exposed	1-2	145	145	135	141	211	211	135	141	140	142	176	196	150	150	M
**C154**	18	29.03.2006	small	mucous spraint	non-exposed	1-2	129	129	135	141	211	211	135	135	142	144	172	180	146	150	F
**C155**	18	29.03.2006	small	jelly	actively exposed	1-2	129	129	135	141	211	211	135	135	142	144	172	180	146	150	F
**D066**	5	30.03.2006	small	jelly	actively exposed	>4	129	145	135	139	211	211	135	141	140	140	176	180	150	150	F
**D067**	5	30.03.2006	medium	jelly	actively exposed	>4	129	145	135	139	211	211	135	141	140	140	176	180	150	150	F
**D069**	5	30.03.2006	large	jelly	actively exposed	3-4	129	145	135	139	211	211	135	141	140	140	176	180	150	150	F
**D074**	8	30.03.2006	large	jelly	actively exposed	>4	145	145	139	141	211	211	135	141	140	142	176	196	150	150	M
**D076**	1	30.03.2006	large	mucous spraint	actively exposed	>4	129	129	135	137	211	211	135	135	140	142	172	180	150	150	F
**D079**	1	30.03.2006	large	mucous spraint	actively exposed	>4	129	129	135	137	211	211	135	135	140	142	172	180	150	150	F
**D084**	10	30.03.2006	small	jelly	non-exposed	>4	129	145	135	139	203	211	135	141	140	142	180	196	150	150	M
**D085**	10	30.03.2006	large	mucous spraint	non-exposed	>4	129	145	135	139	203	211	135	141	140	142	180	196	150	150	M
**D102**	19	30.03.2006	medium	mucous spraint	passively exposed	>4	129	129	135	141	207	211	135	141	142	144	180	180	146	150	F
**D105**	19	30.03.2006	medium	mucous spraint	actively exposed	>4	129	129	135	141	207	211	135	141	142	144	180	180	146	150	F

**D106**	19	30.03.2006	medium	mucous spraint	actively exposed	3-4	129	129	135	141	207	211	135	141	142	144	180	180	146	150	F
**D108**	21	30.03.2006	large	mucous spraint	passively exposed	1-2	145	145	135	141	211	211	135	141	140	142	176	196	150	150	M
**D110**	22	30.03.2006	medium	spraint	non-exposed	1-2	129	145	135	141	211	211	135	141	140	142	176	196	146	150	M
**D113**	19	30.03.2006	small	mucous spraint	actively exposed	3-4	129	129	135	141	207	211	135	141	142	144	180	180	146	150	F
**D141**	17	30.03.2006	large	spraint	non-exposed	>4	145	145	137	139	211	211	135	135	140	142	176	180	146	150	F
**D142**	17	30.03.2006	large	spraint	passively exposed	>4	145	145	137	139	211	211	135	135	140	142	176	180	146	150	F
**D143**	14	30.03.2006	medium	jelly	non-exposed	>4	129	145	137	139	211	211	135	151	140	142	176	180	146	150	M
**D147**	17	30.03.2006	large	spraint	passively exposed	>4	145	145	137	139	211	211	135	135	140	142	176	180	146	150	F
**D148**	17	30.03.2006	large	mucous spraint	passively exposed	>4	145	145	137	139	211	211	135	135	140	142	176	180	146	150	F
**D149**	17	30.03.2006	large	spraint	passively exposed	1-2	145	145	137	139	211	211	135	135	140	142	176	180	146	150	F
**D151**	16	30.03.2006	large	mucous spraint	passively exposed	>4	129	145	137	141	211	211	135	151	140	142	172	176	146	146	F
**D152**	17	30.03.2006	small	spraint	passively exposed	>4	145	145	137	139	211	211	135	135	140	142	176	180	146	150	F
**D153**	16	30.03.2006	large	mucous spraint	passively exposed	>4	129	145	137	141	211	211	135	151	140	142	172	176	146	146	F
**D157**	16	30.03.2006	medium	spraint	passively exposed	>4	129	145	137	141	211	211	135	151	140	142	172	176	146	146	F
**D158**	16	30.03.2006	large	spraint	passively exposed	>4	129	145	137	141	211	211	135	151	140	142	172	176	146	146	F
**D160**	17	30.03.2006	medium	spraint	passively exposed	>4	145	145	137	139	211	211	135	135	140	142	176	180	146	150	F
**DC134**	1	30.03.2006	medium	jelly	actively exposed	>4	129	129	135	137	211	211	135	135	140	142	172	180	150	150	F
**E002**	10	31.03.2006	large	mucous spraint	non-exposed	3-4	129	145	135	139	203	211	135	141	140	142	180	196	150	150	M
**E004**	13	31.03.2006	small	jelly	actively exposed	3-4	129	145	139	141	211	211	135	141	140	142	172	180	150	150	F
**E005**	10	31.03.2006	medium	mucous spraint	actively exposed	3-4	129	145	135	139	203	211	135	141	140	142	180	196	150	150	M
**E014**	10	31.03.2006	large	mucous spraint	actively exposed	>4	129	145	135	139	203	211	135	141	140	142	180	196	150	150	M
**E015**	10	31.03.2006	small	jelly	actively exposed	>4	129	145	135	139	203	211	135	141	140	142	180	196	150	150	M
**E061**	17	31.03.2006	small	jelly	non-exposed	1-2	145	145	137	139	211	211	135	135	140	142	176	180	146	150	F
**E064**	16	31.03.2006	large	mucous spraint	passively exposed	>4	129	145	137	141	211	211	135	151	140	142	172	176	146	146	F
**E065**	17	31.03.2006	medium	mucous spraint	passively exposed	>4	145	145	137	139	211	211	135	135	140	142	176	180	146	150	F
**E068**	16	31.03.2006	large	mucous spraint	passively exposed	1-2	129	145	137	141	211	211	135	151	140	142	172	176	146	146	F
**E076**	17	31.03.2006	medium	mucous spraint	passively exposed	>4	145	145	137	139	211	211	135	135	140	142	176	180	146	150	F
**E084**	9	31.03.2006	large	mucous spraint	actively exposed	3-4	145	145	137	139	211	211	135	141	140	142	176	196	150	150	M
**E091**	7	31.03.2006	large	mucous spraint	non-exposed	1-2	129	147	135	139	211	211	135	135	140	148	176	180	146	150	M
**E092**	8	31.03.2006	medium	jelly	passively exposed	1-2	145	145	139	141	211	211	135	141	140	142	176	196	150	150	M
**E094**	8	31.03.2006	large	mucous spraint	actively exposed	3-4	145	145	139	141	211	211	135	141	140	142	176	196	150	150	M
**E095**	8	31.03.2006	large	jelly	non-exposed	1-2	145	145	139	141	211	211	135	141	140	142	176	196	150	150	M
**E096**	8	31.03.2006	large	jelly	actively exposed	>4	145	145	139	141	211	211	135	141	140	142	176	196	150	150	M
**E097**	7	31.03.2006	medium	mucous spraint	actively exposed	>4	129	147	135	139	211	211	135	135	140	148	176	180	146	150	M
**E098**	7	31.03.2006	large	jelly	actively exposed	>4	129	147	135	139	211	211	135	135	140	148	176	180	146	150	M
**E099**	7	31.03.2006	large	jelly	actively exposed	>4	129	147	135	139	211	211	135	135	140	148	176	180	146	150	M
**E100**	2	31.03.2006	NA	mucous spraint	NA	>4	129	129	135	137	211	211	135	135	140	142	172	196	150	150	M
**E122**	19	31.03.2006	medium	spraint	non-exposed	>4	129	129	135	141	207	211	135	141	142	144	180	180	146	150	F
**E127**	21	31.03.2006	large	mucous spraint	actively exposed	>4	145	145	135	141	211	211	135	141	140	142	176	196	150	150	M
**E128**	15	31.03.2006	small	mucous spraint	passively exposed	1-2	129	129	135	141	207	211	135	141	142	144	180	180	146	150	M
**E133**	19	31.03.2006	small	mucous spraint	actively exposed	3-4	129	129	135	141	207	211	135	141	142	144	180	180	146	150	F
**F003-2**	24	24.04.2007	large	spraint	actively exposed	>4	129	129	137	137	211	211	135	135	140	142	180	196	150	150	F

**F004-2**	25	24.04.2007	small	jelly	passively exposed	3-4	129	145	137	139	203	211	135	141	140	142	176	180	150	150	F
**F008-3**	17	25.04.2007	small	jelly	passively exposed	1-2	145	145	137	139	211	211	135	135	140	142	176	180	146	150	F
**F009-3**	26	25.04.2007	small	spraint	non-exposed	1-2	129	145	137	139	211	211	135	151	140	142	172	176	146	150	M
**F012-3**	26	25.04.2007	small	spraint	non-exposed	3-4	129	145	137	139	211	211	135	151	140	142	172	176	146	150	M
**F014-3**	83	25.04.2007	small	spraint	passively exposed	1-2	145	145	137	139	211	211	135	151	140	142	172	172	146	150	M
**F015-3**	26	25.04.2007	small	jelly	passively exposed	1-2	129	145	137	139	211	211	135	151	140	142	172	176	146	150	M
**F016-4**	16	26.04.2007	medium	mucous spraint	passively exposed	1-2	129	145	137	141	211	211	135	151	140	142	172	176	146	146	F
**F017-3**	17	25.04.2007	small	jelly	passively exposed	1-2	145	145	137	139	211	211	135	135	140	142	176	180	146	150	F
**F018-4**	16	26.04.2007	small	spraint	non-exposed	1-2	129	145	137	141	211	211	135	151	140	142	172	176	146	146	F
**F027-2**	28	24.04.2007	medium	jelly	non-exposed	>4	139	147	137	139	211	211	135	135	148	148	176	176	146	150	M
**F028-2**	28	24.04.2007	small	spraint	actively exposed	>4	139	147	137	139	211	211	135	135	148	148	176	176	146	150	M
**F030-2**	28	24.04.2007	large	jelly	actively exposed	>4	139	147	137	139	211	211	135	135	148	148	176	176	146	150	M
**F031-2**	7	24.04.2007	large	jelly	actively exposed	>4	129	147	135	139	211	211	135	135	140	148	176	180	146	150	M
**F032-2**	7	24.04.2007	small	jelly	non-exposed	>4	129	147	135	139	211	211	135	135	140	148	176	180	146	150	M
**F033-2**	28	24.04.2007	small	mucous spraint	non-exposed	>4	139	147	137	139	211	211	135	135	148	148	176	176	146	150	M
**F035-2**	33	24.04.2007	large	spraint	actively exposed	>4	129	129	135	135	211	211	135	135	140	142	172	172	150	150	F
**F036-2**	28	24.04.2007	medium	spraint	actively exposed	>4	139	147	137	139	211	211	135	135	148	148	176	176	146	150	M
**F037-2**	28	24.04.2007	medium	mucous spraint	actively exposed	>4	139	147	137	139	211	211	135	135	148	148	176	176	146	150	M
**F038-1**	7	23.04.2007	small	jelly	actively exposed	>4	129	147	135	139	211	211	135	135	140	148	176	180	146	150	M
**F039-1**	25	23.04.2007	small	mucous spraint	actively exposed	1-2	129	145	137	139	203	211	135	141	140	142	176	180	150	150	F
**F041-2**	1	24.04.2007	large	jelly	actively exposed	>4	129	129	135	137	211	211	135	135	140	142	172	180	150	150	F
**F042-2**	24	24.04.2007	large	mucous spraint	non-exposed	>4	129	129	137	137	211	211	135	135	140	142	180	196	150	150	F
**F045-1**	33	23.04.2007	medium	mucous spraint	actively exposed	>4	129	129	135	135	211	211	135	135	140	142	172	172	150	150	F
**F047-1**	7	23.04.2007	small	jelly	actively exposed	>4	129	147	135	139	211	211	135	135	140	148	176	180	146	150	M
**F049-1**	31	23.04.2007	large	jelly	passively exposed	>4	129	129	137	137	211	211	135	135	140	142	180	196	150	150	M
**F052-1**	24	23.04.2007	medium	spraint	passively exposed	1-2	129	129	137	137	211	211	135	135	140	142	180	196	150	150	F
**F053-1**	28	23.04.2007	medium	spraint	passively exposed	1-2	139	147	137	139	211	211	135	135	148	148	176	176	146	150	M
**F054-2**	5	24.04.2007	small	spraint	passively exposed	1-2	129	145	135	139	211	211	135	141	140	140	176	180	150	150	F
**F055-2**	32	24.04.2007	medium	mucous spraint	non-exposed	1-2	129	129	135	139	211	211	135	141	140	142	176	196	150	150	M
**F056-2**	28	24.04.2007	large	spraint	passively exposed	3-4	139	147	137	139	211	211	135	135	148	148	176	176	146	150	M
**F057-2**	7	24.04.2007	large	spraint	passively exposed	3-4	129	147	135	139	211	211	135	135	140	148	176	180	146	150	M
**F058-2**	28	24.04.2007	large	mucous spraint	non-exposed	>4	139	147	137	139	211	211	135	135	148	148	176	176	146	150	M
**F061-2**	7	24.04.2007	large	mucous spraint	passively exposed	3-4	129	147	135	139	211	211	135	135	140	148	176	180	146	150	M
**F062-2**	28	24.04.2007	medium	mucous spraint	non-exposed	3-4	139	147	137	139	211	211	135	135	148	148	176	176	146	150	M
**F064-2**	28	24.04.2007	large	mucous spraint	passively exposed	3-4	139	147	137	139	211	211	135	135	148	148	176	176	146	150	M
**F065-2**	33	24.04.2007	large	spraint	actively exposed	1-2	129	129	135	135	211	211	135	135	140	142	172	172	150	150	F
**F068-3**	34	25.04.2007	large	spraint	non-exposed	>4	129	145	137	139	211	211	135	151	140	142	172	196	150	150	M
**F069-3**	5	25.04.2007	large	jelly	passively exposed	>4	129	145	135	139	211	211	135	141	140	140	176	180	150	150	F
**F074-2**	25	24.04.2007	small	jelly	actively exposed	3-4	129	145	137	139	203	211	135	141	140	142	176	180	150	150	F
**F075-2**	27	24.04.2007	small	mucous spraint	non-exposed	1-2	129	145	135	139	203	211	135	135	140	142	172	176	150	150	F
**F076-1**	23	23.04.2007	medium	spraint	passively exposed	1-2	129	145	139	141	211	211	135	135	140	142	172	180	150	150	M
**F077-1**	36	23.04.2007	small	spraint	passively exposed	1-2	129	129	135	139	203	211	135	135	140	142	172	180	150	150	M

**F081-2**	36	24.04.2007	medium	spraint	passively exposed	1-2	129	129	135	139	203	211	135	135	140	142	172	180	150	150	M
**F087-5**	35	27.04.2007	large	mucous spraint	non-exposed	1-2	129	129	137	139	207	211	141	141	140	142	180	180	146	150	F
**F090-3**	35	25.04.2007	small	jelly	actively exposed	1-2	129	129	137	139	207	211	141	141	140	142	180	180	146	150	F
**F092-3**	35	25.04.2007	medium	spraint	passively exposed	1-2	129	129	137	139	207	211	141	141	140	142	180	180	146	150	F
**F093-5**	35	27.04.2007	small	spraint	passively exposed	1-2	129	129	137	139	207	211	141	141	140	142	180	180	146	150	F
**F094-5**	36	27.04.2007	small	jelly	passively exposed	1-2	129	129	135	139	203	211	135	135	140	142	172	180	150	150	M
**F099-5**	36	27.04.2007	small	spraint	passively exposed	1-2	129	129	135	139	203	211	135	135	140	142	172	180	150	150	M
**F102-1**	10	23.04.2007	small	spraint	actively exposed	3-4	129	145	135	139	203	211	135	141	140	142	180	196	150	150	M
**F105-3**	41	25.04.2007	small	spraint	non-exposed	1-2	129	145	139	141	211	211	135	141	140	142	172	180	146	150	F
**F108-2**	28	24.04.2007	large	jelly	actively exposed	>4	139	147	137	139	211	211	135	135	148	148	176	176	146	150	M
**F109-2**	10	24.04.2007	small	mucous spraint	non-exposed	3-4	129	145	135	139	203	211	135	141	140	142	180	196	150	150	M
**F113-1**	11	23.04.2007	small	spraint	actively exposed	1-2	129	145	135	141	203	211	141	141	140	142	176	180	150	150	M
**F114-2**	1	24.04.2007	small	jelly	actively exposed	>4	129	129	135	137	211	211	135	135	140	142	172	180	150	150	F
**F117-2**	30	24.04.2007	medium	mucous spraint	actively exposed	>4	129	147	139	139	211	211	135	141	142	148	180	180	146	150	F
**F118-2**	30	24.04.2007	medium	mucous spraint	non-exposed	>4	129	147	139	139	211	211	135	141	142	148	180	180	146	150	F
**F119-2**	41	24.04.2007	small	mucous spraint	passively exposed	1-2	129	145	139	141	211	211	135	141	140	142	172	180	146	150	F
**F120-2**	30	24.04.2007	large	jelly	actively exposed	>4	129	147	139	139	211	211	135	141	142	148	180	180	146	150	F
**F123-2**	11	24.04.2007	large	spraint	passively exposed	1-2	129	145	135	141	203	211	141	141	140	142	176	180	150	150	M
**F125-2**	7	24.04.2007	large	mucous spraint	passively exposed	1-2	129	147	135	139	211	211	135	135	140	148	176	180	146	150	M
**G001-4**	7	26.04.2007	large	mucous spraint	actively exposed	3-4	129	147	135	139	211	211	135	135	140	148	176	180	146	150	M
**G003-5**	7	27.04.2007	medium	spraint	passively exposed	1-2	129	147	135	139	211	211	135	135	140	148	176	180	146	150	M
**G006-3**	28	25.04.2007	medium	mucous spraint	actively exposed	>4	139	147	137	139	211	211	135	135	148	148	176	176	146	150	M
**G010-4**	7	26.04.2007	large	mucous spraint	actively exposed	>4	129	147	135	139	211	211	135	135	140	148	176	180	146	150	M
**G011-3**	6	25.04.2007	large	spraint	passively exposed	3-4	129	145	135	139	211	211	141	151	140	142	172	176	150	150	F
**G013-4**	13	26.04.2007	small	jelly	non-exposed	>4	129	145	139	141	211	211	135	141	140	142	172	180	150	150	F
**G014-3**	10	25.04.2007	medium	mucous spraint	non-exposed	>4	129	145	135	139	203	211	135	141	140	142	180	196	150	150	M
**G020-4**	10	26.04.2007	small	jelly	passively exposed	1-2	129	145	135	139	203	211	135	141	140	142	180	196	150	150	M
**G021-3**	40	25.04.2007	medium	jelly	actively exposed	>4	129	145	135	139	203	211	135	141	140	142	176	180	146	150	M
**G023-3**	7	25.04.2007	small	mucous spraint	actively exposed	>4	129	147	135	139	211	211	135	135	140	148	176	180	146	150	M
**G028-3**	34	25.04.2007	medium	spraint	passively exposed	>4	129	145	137	139	211	211	135	151	140	142	172	196	150	150	M
**G030-3**	28	25.04.2007	small	mucous spraint	actively exposed	>4	139	147	137	139	211	211	135	135	148	148	176	176	146	150	M
**G032-4**	23	26.04.2007	small	spraint	actively exposed	1-2	129	145	139	141	211	211	135	135	140	142	172	180	150	150	M
**G033-4**	23	26.04.2007	small	jelly	passively exposed	3-4	129	145	139	141	211	211	135	135	140	142	172	180	150	150	M
**G037-3**	35	25.04.2007	small	jelly	passively exposed	1-2	129	129	137	139	207	211	141	141	140	142	180	180	146	150	F
**G042-4**	23	26.04.2007	small	jelly	passively exposed	3-4	129	145	139	141	211	211	135	135	140	142	172	180	150	150	M
**G043-5**	29	27.04.2007	large	mucous spraint	passively exposed	>4	129	147	139	139	211	211	135	135	140	140	176	180	150	150	F
**G044-4**	10	26.04.2007	medium	mucous spraint	passively exposed	3-4	129	145	135	139	203	211	135	141	140	142	180	196	150	150	M
**G045-4**	29	26.04.2007	medium	spraint	passively exposed	>4	129	147	139	139	211	211	135	135	140	140	176	180	150	150	F
**G046-4**	13	26.04.2007	large	spraint	non-exposed	3-4	129	145	139	141	211	211	135	141	140	142	172	180	150	150	F
**G050-5**	13	27.04.2007	small	jelly	passively exposed	3-4	129	145	139	141	211	211	135	141	140	142	172	180	150	150	F
**G051-5**	7	27.04.2007	small	spraint	actively exposed	3-4	129	147	135	139	211	211	135	135	140	148	176	180	146	150	M
**G063-4**	37	26.04.2007	large	mucous spraint	passively exposed	>4	129	145	139	139	211	211	135	135	140	142	180	196	150	150	F

**G066-3**	24	25.04.2007	large	jelly	passively exposed	3-4	129	129	137	137	211	211	135	135	140	142	180	196	150	150	F
**G067-3**	7	25.04.2007	medium	jelly	passively exposed	1-2	129	147	135	139	211	211	135	135	140	148	176	180	146	150	M
**G069-3**	28	25.04.2007	medium	spraint	passively exposed	3-4	139	147	137	139	211	211	135	135	148	148	176	176	146	150	M
**G071-3**	33	25.04.2007	large	mucous spraint	non-exposed	3-4	129	129	135	135	211	211	135	135	140	142	172	172	150	150	F
**G075-3**	39	25.04.2007	small	jelly	non-exposed	3-4	129	145	135	139	203	211	135	141	140	142	180	196	146	150	M
**G079-3**	33	25.04.2007	small	spraint	passively exposed	3-4	129	129	135	135	211	211	135	135	140	142	172	172	150	150	F
**G083-3**	11	25.04.2007	large	jelly	actively exposed	>4	129	145	135	141	203	211	141	141	140	142	176	180	150	150	M
**G086-4**	33	26.04.2007	large	mucous spraint	non-exposed	>4	129	129	135	135	211	211	135	135	140	142	172	172	150	150	F
**G087-3**	25	25.04.2007	small	jelly	actively exposed	3-4	129	145	137	139	203	211	135	141	140	142	176	180	150	150	F
**G089-4**	28	26.04.2007	small	spraint	passively exposed	3-4	139	147	137	139	211	211	135	135	148	148	176	176	146	150	M
**G092-4**	33	26.04.2007	large	mucous spraint	non-exposed	>4	129	129	135	135	211	211	135	135	140	142	172	172	150	150	F
**G096-4**	34	26.04.2007	large	jelly	non-exposed	>4	129	145	137	139	211	211	135	151	140	142	172	196	150	150	M
**G097-3**	7	25.04.2007	medium	jelly	actively exposed	>4	129	147	135	139	211	211	135	135	140	148	176	180	146	150	M
**G098-5**	6	27.04.2007	medium	mucous spraint	non-exposed	>4	129	145	135	139	211	211	141	151	140	142	172	176	150	150	F
**G099-3**	1	25.04.2007	small	jelly	actively exposed	>4	129	129	135	137	211	211	135	135	140	142	172	180	150	150	F
**G105-3**	24	25.04.2007	large	mucous spraint	actively exposed	>4	129	129	137	137	211	211	135	135	140	142	180	196	150	150	F
**G108-5**	34	27.04.2007	large	spraint	non-exposed	>4	129	145	137	139	211	211	135	151	140	142	172	196	150	150	M
**G110-5**	34	27.04.2007	medium	mucous spraint	non-exposed	>4	129	145	137	139	211	211	135	151	140	142	172	196	150	150	M
**G111-4**	2	26.04.2007	medium	spraint	passively exposed	>4	129	129	135	137	211	211	135	135	140	142	172	196	150	150	M
**G114-3**	28	25.04.2007	large	mucous spraint	actively exposed	>4	139	147	137	139	211	211	135	135	148	148	176	176	146	150	M
**G116-4**	5	26.04.2007	small	spraint	actively exposed	3-4	129	145	135	139	211	211	135	141	140	140	176	180	150	150	F
**H003-5**	24	27.04.2007	large	mucous spraint	passively exposed	3-4	129	129	137	137	211	211	135	135	140	142	180	196	150	150	F
**H007-4**	28	26.04.2007	small	jelly	actively exposed	>4	139	147	137	139	211	211	135	135	148	148	176	176	146	150	M
**H008-5**	28	27.04.2007	medium	mucous spraint	actively exposed	>4	139	147	137	139	211	211	135	135	148	148	176	176	146	150	M
**H009-5**	28	27.04.2007	small	mucous spraint	actively exposed	>4	139	147	137	139	211	211	135	135	148	148	176	176	146	150	M
**H011-5**	39	27.04.2007	large	jelly	passively exposed	>4	129	145	135	139	203	211	135	141	140	142	180	196	146	150	M
**H012-5**	24	27.04.2007	medium	spraint	passively exposed	3-4	129	129	137	137	211	211	135	135	140	142	180	196	150	150	F
**H013-4**	28	26.04.2007	small	mucous spraint	actively exposed	>4	139	147	137	139	211	211	135	135	148	148	176	176	146	150	M
**H016-4**	24	26.04.2007	small	mucous spraint	actively exposed	>4	129	129	137	137	211	211	135	135	140	142	180	196	150	150	F
**H022-5**	33	27.04.2007	small	spraint	passively exposed	1-2	129	129	135	135	211	211	135	135	140	142	172	172	150	150	F
**H025-5**	33	27.04.2007	large	mucous spraint	passively exposed	1-2	129	129	135	135	211	211	135	135	140	142	172	172	150	150	F
**H026-5**	38	27.04.2007	large	mucous spraint	non-exposed	>4	129	145	135	139	211	211	141	151	140	142	172	176	146	150	F
**H029-5**	24	27.04.2007	small	jelly	non-exposed	>4	129	129	137	137	211	211	135	135	140	142	180	196	150	150	F
**H030-4**	7	26.04.2007	small	jelly	actively exposed	>4	129	147	135	139	211	211	135	135	140	148	176	180	146	150	M
**H035-5**	34	27.04.2007	medium	mucous spraint	actively exposed	>4	129	145	137	139	211	211	135	151	140	142	172	196	150	150	M
**H037-4**	28	26.04.2007	medium	spraint	actively exposed	>4	139	147	137	139	211	211	135	135	148	148	176	176	146	150	M
**H038-4**	24	26.04.2007	small	jelly	non-exposed	>4	129	129	137	137	211	211	135	135	140	142	180	196	150	150	F
**H039-5**	6	27.04.2007	large	mucous spraint	actively exposed	>4	129	145	135	139	211	211	141	151	140	142	172	176	150	150	F
**H044-4**	24	26.04.2007	medium	spraint	actively exposed	>4	129	129	137	137	211	211	135	135	140	142	180	196	150	150	F
**H046-4**	24	26.04.2007	small	mucous spraint	actively exposed	>4	129	129	137	137	211	211	135	135	140	142	180	196	150	150	F
**H048-4**	28	26.04.2007	small	mucous spraint	actively exposed	>4	139	147	137	139	211	211	135	135	148	148	176	176	146	150	M
**H049-4**	34	26.04.2007	small	mucous spraint	passively exposed	3-4	129	145	137	139	211	211	135	151	140	142	172	196	150	150	M

**H050-4**	6	26.04.2007	medium	spraint	passively exposed	>4	129	145	135	139	211	211	141	151	140	142	172	176	150	150	F
**H051-4**	6	26.04.2007	large	mucous spraint	passively exposed	3-4	129	145	135	139	211	211	141	151	140	142	172	176	150	150	F
**H052-4**	7	26.04.2007	small	mucous spraint	passively exposed	3-4	129	147	135	139	211	211	135	135	140	148	176	180	146	150	M
**H054-4**	6	26.04.2007	small	mucous spraint	passively exposed	3-4	129	145	135	139	211	211	141	151	140	142	172	176	150	150	F
**H059-5**	28	27.04.2007	large	jelly	actively exposed	>4	139	147	137	139	211	211	135	135	148	148	176	176	146	150	M
**H061-5**	28	27.04.2007	small	mucous spraint	actively exposed	>4	139	147	137	139	211	211	135	135	148	148	176	176	146	150	M
**H064-5**	28	27.04.2007	small	spraint	passively exposed	>4	139	147	137	139	211	211	135	135	148	148	176	176	146	150	M
**K013**	7	26.05.2008	medium	jelly	passively exposed	3-4	129	147	135	139	211	211	135	135	140	148	176	180	146	150	M
**K034**	50	26.05.2008	large	spraint	non-exposed	1-2	129	145	135	137	211	215	135	135	140	142	176	180	150	150	M
**K070**	34	26.05.2008	small	spraint	actively exposed	3-4	129	145	137	139	211	211	135	151	140	142	172	196	150	150	M
**K074**	6	26.05.2008	medium	spraint	actively exposed	>4	129	145	135	139	211	211	141	151	140	142	172	176	150	150	F
**K086**	30	26.05.2008	small	mucous spraint	passively exposed	3-4	129	147	139	139	211	211	135	141	142	148	180	180	146	150	F
**K101**	44	26.05.2008	medium	mucous spraint	actively exposed	3-4	129	129	137	139	207	211	135	141	140	142	180	180	146	150	F
**K105**	43	26.05.2008	large	spraint	passively exposed	3-4	129	129	137	137	211	211	141	141	140	142	172	172	146	150	F
**K110**	48	26.05.2008	small	spraint	actively exposed	3-4	147	147	135	139	211	211	135	135	142	148	172	176	146	150	M
**K111**	32	26.05.2008	medium	jelly	actively exposed	3-4	129	129	135	139	211	211	135	141	140	142	176	196	150	150	M
**K115**	7	26.05.2008	large	jelly	non-exposed	3-4	129	147	135	139	211	211	135	135	140	148	176	180	146	150	M
**K124**	16	26.05.2008	medium	jelly	passively exposed	3-4	129	145	137	141	211	211	135	151	140	142	172	176	146	146	F
**K128**	42	26.05.2008	large	spraint	non-exposed	>4	129	139	135	139	211	211	135	141	140	142	176	180	150	150	M
**K129**	32	26.05.2008	large	spraint	passively exposed	>4	129	129	135	139	211	211	135	141	140	142	176	196	150	150	M
**K130**	5	26.05.2008	small	mucous spraint	non-exposed	>4	129	145	135	139	211	211	135	141	140	140	176	180	150	150	F
**K141**	52	26.05.2008	medium	spraint	actively exposed	>4	129	145	135	139	211	211	135	141	140	142	176	180	150	150	F
**L014**	48	27.05.2008	medium	spraint	actively exposed	1-2	147	147	135	139	211	211	135	135	142	148	172	176	146	150	M
**L017**	50	27.05.2008	medium	spraint	actively exposed	3-4	129	145	135	137	211	215	135	135	140	142	176	180	150	150	M
**L018**	44	27.05.2008	small	spraint	actively exposed	3-4	129	129	137	139	207	211	135	141	140	142	180	180	146	150	F
**L053**	27	27.05.2008	large	jelly	passively exposed	1-2	129	145	135	139	203	211	135	135	140	142	172	176	150	150	F
**L058**	10	27.05.2008	large	jelly	actively exposed	>4	129	145	135	139	203	211	135	141	140	142	180	196	150	150	M
**L098**	49	27.05.2008	large	mucous spraint	actively exposed	3-4	129	145	139	139	207	211	135	141	140	140	180	180	150	150	F
**L102**	32	27.05.2008	medium	jelly	passively exposed	3-4	129	129	135	139	211	211	135	141	140	142	176	196	150	150	M
**L103**	32	27.05.2008	large	spraint	passively exposed	3-4	129	129	135	139	211	211	135	141	140	142	176	196	150	150	M
**L104**	32	27.05.2008	medium	jelly	actively exposed	1-2	129	129	135	139	211	211	135	141	140	142	176	196	150	150	M
**L105**	32	27.05.2008	large	spraint	non-exposed	>4	129	129	135	139	211	211	135	141	140	142	176	196	150	150	M
**L108**	34	27.05.2008	medium	mucous spraint	passively exposed	>4	129	145	137	139	211	211	135	151	140	142	172	196	150	150	M
**L109**	34	27.05.2008	large	mucous spraint	actively exposed	3-4	129	145	137	139	211	211	135	151	140	142	172	196	150	150	M
**L110**	6	27.05.2008	small	spraint	non-exposed	>4	129	145	135	139	211	211	141	151	140	142	172	176	150	150	F
**L111**	6	27.05.2008	small	jelly	passively exposed	3-4	129	145	135	139	211	211	141	151	140	142	172	176	150	150	F
**L112**	7	27.05.2008	medium	mucous spraint	passively exposed	3-4	129	147	135	139	211	211	135	135	140	148	176	180	146	150	M
**L113**	5	27.05.2008	medium	jelly	actively exposed	1-2	129	145	135	139	211	211	135	141	140	140	176	180	150	150	F
**M011**	16	28.05.2008	large	jelly	non-exposed	1-2	129	145	137	141	211	211	135	151	140	142	172	176	146	146	F
**M012**	16	28.05.2008	medium	spraint	non-exposed	3-4	129	145	137	141	211	211	135	151	140	142	172	176	146	146	F
**M019**	7	28.05.2008	small	jelly	non-exposed	1-2	129	147	135	139	211	211	135	135	140	148	176	180	146	150	M
**M039**	50	28.05.2008	small	spraint	passively exposed	3-4	129	145	135	137	211	215	135	135	140	142	176	180	150	150	M

**M041**	50	28.05.2008	large	spraint	passively exposed	3-4	129	145	135	137	211	215	135	135	140	142	176	180	150	150	M
**M042**	50	28.05.2008	large	spraint	non-exposed	3-4	129	145	135	137	211	215	135	135	140	142	176	180	150	150	M
**M045**	50	28.05.2008	medium	mucous spraint	non-exposed	3-4	129	145	135	137	211	215	135	135	140	142	176	180	150	150	M
**M066**	34	28.05.2008	medium	spraint	passively exposed	>4	129	145	137	139	211	211	135	151	140	142	172	196	150	150	M
**M069**	6	28.05.2008	small	jelly	passively exposed	>4	129	145	135	139	211	211	141	151	140	142	172	176	150	150	F
**M071**	32	28.05.2008	small	jelly	actively exposed	1-2	129	129	135	139	211	211	135	141	140	142	176	196	150	150	M
**M072**	34	28.05.2008	large	spraint	non-exposed	>4	129	145	137	139	211	211	135	151	140	142	172	196	150	150	M
**M075**	42	28.05.2008	medium	mucous spraint	actively exposed	3-4	129	139	135	139	211	211	135	141	140	142	176	180	150	150	M
**M076**	32	28.05.2008	large	spraint	passively exposed	3-4	129	129	135	139	211	211	135	141	140	142	176	196	150	150	M
**M077**	32	28.05.2008	small	mucous spraint	actively exposed	>4	129	129	135	139	211	211	135	141	140	142	176	196	150	150	M
**M078**	32	28.05.2008	large	mucous spraint	passively exposed	>4	129	129	135	139	211	211	135	141	140	142	176	196	150	150	M
**M079**	32	28.05.2008	small	jelly	passively exposed	>4	129	129	135	139	211	211	135	141	140	142	176	196	150	150	M
**M080**	32	28.05.2008	small	jelly	actively exposed	1-2	129	129	135	139	211	211	135	141	140	142	176	196	150	150	M
**M083**	10	28.05.2008	medium	mucous spraint	actively exposed	>4	129	145	135	139	203	211	135	141	140	142	180	196	150	150	M
**M084**	27	28.05.2008	small	jelly	passively exposed	3-4	129	145	135	139	203	211	135	135	140	142	172	176	150	150	F
**M091**	10	28.05.2008	large	jelly	actively exposed	>4	129	145	135	139	203	211	135	141	140	142	180	196	150	150	M
**M092**	10	28.05.2008	small	jelly	actively exposed	>4	129	145	135	139	203	211	135	141	140	142	180	196	150	150	M
**M094**	30	28.05.2008	medium	spraint	non-exposed	3-4	129	147	139	139	211	211	135	141	142	148	180	180	146	150	F
**M098**	30	28.05.2008	small	spraint	actively exposed	3-4	129	147	139	139	211	211	135	141	142	148	180	180	146	150	F
**N007**	45	29.05.2008	medium	spraint	passively exposed	3-4	129	129	139	141	211	211	135	141	140	140	176	180	150	150	F
**N010**	27	29.05.2008	large	spraint	passively exposed	3-4	129	145	135	139	203	211	135	135	140	142	172	176	150	150	F
**N015**	30	29.05.2008	medium	spraint	non-exposed	3-4	129	147	139	139	211	211	135	141	142	148	180	180	146	150	F
**N020**	30	29.05.2008	small	mucous spraint	actively exposed	3-4	129	147	139	139	211	211	135	141	142	148	180	180	146	150	F
**N032**	48	29.05.2008	medium	spraint	actively exposed	3-4	147	147	135	139	211	211	135	135	142	148	172	176	146	150	M
**N033**	48	29.05.2008	small	spraint	actively exposed	3-4	147	147	135	139	211	211	135	135	142	148	172	176	146	150	M
**N034**	49	29.05.2008	small	mucous spraint	actively exposed	>4	129	145	139	139	207	211	135	141	140	140	180	180	150	150	F
**N035**	50	29.05.2008	small	mucous spraint	actively exposed	3-4	129	145	135	137	211	215	135	135	140	142	176	180	150	150	M
**N036**	32	29.05.2008	small	mucous spraint	non-exposed	3-4	129	129	135	139	211	211	135	141	140	142	176	196	150	150	M
**N037**	32	29.05.2008	large	mucous spraint	passively exposed	3-4	129	129	135	139	211	211	135	141	140	142	176	196	150	150	M
**N038**	32	29.05.2008	large	mucous spraint	passively exposed	3-4	129	129	135	139	211	211	135	141	140	142	176	196	150	150	M
**N039**	32	29.05.2008	small	jelly	non-exposed	1-2	129	129	135	139	211	211	135	141	140	142	176	196	150	150	M
**N041**	32	29.05.2008	small	jelly	actively exposed	1-2	129	129	135	139	211	211	135	141	140	142	176	196	150	150	M
**N042**	32	29.05.2008	medium	jelly	non-exposed	>4	129	129	135	139	211	211	135	141	140	142	176	196	150	150	M
**N043**	51	29.05.2008	large	jelly	passively exposed	>4	129	145	135	139	211	211	135	141	140	140	176	180	150	150	M
**N044**	34	29.05.2008	small	jelly	passively exposed	>4	129	145	137	139	211	211	135	151	140	142	172	196	150	150	M
**N045**	42	29.05.2008	large	spraint	non-exposed	>4	129	139	135	139	211	211	135	141	140	142	176	180	150	150	M
**N049**	47	29.05.2008	large	mucous spraint	non-exposed	>4	139	145	137	139	211	211	135	141	140	142	176	180	146	150	M
**N052**	6	29.05.2008	large	jelly	passively exposed	3-4	129	145	135	139	211	211	141	151	140	142	172	176	150	150	F
**N054**	7	29.05.2008	small	jelly	passively exposed	>4	129	147	135	139	211	211	135	135	140	148	176	180	146	150	M
**N055**	42	29.05.2008	small	mucous spraint	non-exposed	>4	129	139	135	139	211	211	135	141	140	142	176	180	150	150	M
**N082**	50	29.05.2008	large	spraint	passively exposed	3-4	129	145	135	137	211	215	135	135	140	142	176	180	150	150	M
**N096**	6	29.05.2008	small	jelly	non-exposed	1-2	129	145	135	139	211	211	141	151	140	142	172	176	150	150	F

**O007**	50	30.05.2008	large	jelly	actively exposed	3-4	129	145	135	137	211	215	135	135	140	142	176	180	150	150	M
**O008**	50	30.05.2008	small	jelly	actively exposed	3-4	129	145	135	137	211	215	135	135	140	142	176	180	150	150	M
**O016**	50	30.05.2008	small	mucous spraint	actively exposed	3-4	129	145	135	137	211	215	135	135	140	142	176	180	150	150	M
**O031**	16	30.05.2008	medium	spraint	actively exposed	1-2	129	145	137	141	211	211	135	151	140	142	172	176	146	146	F
**O032**	21	30.05.2008	large	jelly	passively exposed	1-2	145	145	135	141	211	211	135	141	140	142	176	196	150	150	M
**O039**	46	30.05.2008	medium	mucous spraint	non-exposed	1-2	129	129	139	141	211	211	141	141	140	142	176	180	150	150	F
**O041**	30	30.05.2008	small	spraint	non-exposed	3-4	129	147	139	139	211	211	135	141	142	148	180	180	146	150	F
**O054**	27	30.05.2008	medium	spraint	NA	1-2	129	145	135	139	203	211	135	135	140	142	172	176	150	150	F
**O059**	32	30.05.2008	small	spraint	passively exposed	3-4	129	129	135	139	211	211	135	141	140	142	176	196	150	150	M
**O060**	23	30.05.2008	medium	jelly	actively exposed	>4	129	145	139	141	211	211	135	135	140	142	172	180	150	150	M
**O063**	7	30.05.2008	medium	jelly	non-exposed	>4	129	147	135	139	211	211	135	135	140	148	176	180	146	150	M
**O064**	32	30.05.2008	small	mucous spraint	passively exposed	>4	129	129	135	139	211	211	135	141	140	142	176	196	150	150	M
**O065**	32	30.05.2008	small	jelly	passively exposed	3-4	129	129	135	139	211	211	135	141	140	142	176	196	150	150	M
**O066**	32	30.05.2008	medium	jelly	actively exposed	>4	129	129	135	139	211	211	135	141	140	142	176	196	150	150	M
**O067**	32	30.05.2008	small	spraint	passively exposed	>4	129	129	135	139	211	211	135	141	140	142	176	196	150	150	M
**O068**	32	30.05.2008	small	mucous spraint	non-exposed	>4	129	129	135	139	211	211	135	141	140	142	176	196	150	150	M
**O071**	6	30.05.2008	large	spraint	actively exposed	>4	129	145	135	139	211	211	141	151	140	142	172	176	150	150	F
**O074**	6	30.05.2008	small	mucous spraint	actively exposed	>4	129	145	135	139	211	211	141	151	140	142	172	176	150	150	F
**O078**	7	30.05.2008	medium	jelly	non-exposed	>4	129	147	135	139	211	211	135	135	140	148	176	180	146	150	M
**P003**	42	22.03.2010	medium	spraint	non-exposed	3-4	129	139	135	139	211	211	135	141	140	142	176	180	150	150	M
**P005**	45	22.03.2010	large	mucous spraint	non-exposed	1-2	129	129	139	141	211	211	135	141	140	140	176	180	150	150	F
**P029**	21	22.03.2010	medium	mucous spraint	non-exposed	1-2	145	145	135	141	211	211	135	141	140	142	176	196	150	150	M
**P030**	61	22.03.2010	large	mucous spraint	passively exposed	3-4	129	145	137	139	207	211	135	141	140	142	180	196	146	150	M
**P032**	44	22.03.2010	large	spraint	passively exposed	1-2	129	129	137	139	207	211	135	141	140	142	180	180	146	150	F
**P033**	57	22.03.2010	large	mucous spraint	non-exposed	3-4	129	147	139	139	211	211	135	135	140	142	172	172	146	150	F
**P034**	61	22.03.2010	small	spraint	non-exposed	1-2	129	145	137	139	207	211	135	141	140	142	180	196	146	150	M
**P045**	5	22.03.2010	small	jelly	actively exposed	1-2	129	145	135	139	211	211	135	141	140	140	176	180	150	150	F
**P048**	5	22.03.2010	medium	mucous spraint	non-exposed	3-4	129	145	135	139	211	211	135	141	140	140	176	180	150	150	F
**P051**	34	22.03.2010	medium	spraint	passively exposed	>4	129	145	137	139	211	211	135	151	140	142	172	196	150	150	M
**P055**	5	22.03.2010	small	jelly	actively exposed	3-4	129	145	135	139	211	211	135	141	140	140	176	180	150	150	F
**P057**	56	22.03.2010	small	jelly	actively exposed	3-4	129	145	137	139	211	211	141	151	140	142	180	196	150	150	M
**P059**	34	22.03.2010	large	spraint	passively exposed	>4	129	145	137	139	211	211	135	151	140	142	172	196	150	150	M
**P060**	34	22.03.2010	large	mucous spraint	passively exposed	>4	129	145	137	139	211	211	135	151	140	142	172	196	150	150	M
**P069**	34	22.03.2010	small	spraint	non-exposed	1-2	129	145	137	139	211	211	135	151	140	142	172	196	150	150	M
**P072**	34	22.03.2010	medium	spraint	non-exposed	1-2	129	145	137	139	211	211	135	151	140	142	172	196	150	150	M
**P073**	58	22.03.2010	medium	spraint	passively exposed	1-2	129	145	135	137	211	211	135	135	140	142	176	180	150	150	F
**P074**	1	22.03.2010	large	mucous spraint	passively exposed	1-2	129	129	135	137	211	211	135	135	140	142	172	180	150	150	F
**P080**	34	22.03.2010	large	mucous spraint	non-exposed	1-2	129	145	137	139	211	211	135	151	140	142	172	196	150	150	M
**P085**	57	22.03.2010	medium	jelly	non-exposed	3-4	129	147	139	139	211	211	135	135	140	142	172	172	146	150	F
**P091**	56	22.03.2010	medium	mucous spraint	non-exposed	>4	129	145	137	139	211	211	141	151	140	142	180	196	150	150	M
**P092**	56	22.03.2010	large	spraint	passively exposed	3-4	129	145	137	139	211	211	141	151	140	142	180	196	150	150	M
**P094**	59	22.03.2010	medium	spraint	actively exposed	3-4	129	129	137	139	211	215	135	141	140	142	172	180	150	150	M

**P102**	54	22.03.2010	medium	mucous spraint	actively exposed	1-2	145	145	137	141	211	211	135	141	140	142	180	196	150	150	F
**P104**	53	22.03.2010	small	jelly	non-exposed	3-4	145	145	135	137	211	211	135	141	140	142	176	180	150	150	F
**P107**	62	22.03.2010	small	jelly	passively exposed	3-4	129	145	137	139	211	215	135	141	140	142	176	180	150	150	M
**P109**	17	22.03.2010	medium	spraint	non-exposed	1-2	145	145	137	139	211	211	135	135	140	142	176	180	146	150	F
**P117**	5	22.03.2010	medium	mucous spraint	passively exposed	>4	129	145	135	139	211	211	135	141	140	140	176	180	150	150	F
**Q002**	44	23.03.2010	large	spraint	non-exposed	1-2	129	129	137	139	207	211	135	141	140	142	180	180	146	150	F
**Q014**	45	23.03.2010	medium	mucous spraint	non-exposed	1-2	129	129	139	141	211	211	135	141	140	140	176	180	150	150	F
**Q022**	46	23.03.2010	large	mucous spraint	passively exposed	1-2	129	129	139	141	211	211	141	141	140	142	176	180	150	150	F
**Q023**	42	23.03.2010	large	spraint	passively exposed	3-4	129	139	135	139	211	211	135	141	140	142	176	180	150	150	M
**Q026**	45	23.03.2010	small	jelly	non-exposed	3-4	129	129	139	141	211	211	135	141	140	140	176	180	150	150	F
**Q027**	45	23.03.2010	medium	spraint	actively exposed	1-2	129	129	139	141	211	211	135	141	140	140	176	180	150	150	F
**Q028**	46	23.03.2010	small	jelly	actively exposed	1-2	129	129	139	141	211	211	141	141	140	142	176	180	150	150	F
**Q033**	45	23.03.2010	medium	spraint	passively exposed	3-4	129	129	139	141	211	211	135	141	140	140	176	180	150	150	F
**Q035**	46	23.03.2010	small	spraint	actively exposed	1-2	129	129	139	141	211	211	141	141	140	142	176	180	150	150	F
**Q040**	1	23.03.2010	medium	mucous spraint	passively exposed	1-2	129	129	135	137	211	211	135	135	140	142	172	180	150	150	F
**Q042**	1	23.03.2010	small	jelly	non-exposed	1-2	129	129	135	137	211	211	135	135	140	142	172	180	150	150	F
**Q053**	1	23.03.2010	medium	mucous spraint	passively exposed	>4	129	129	135	137	211	211	135	135	140	142	172	180	150	150	F
**Q060**	34	23.03.2010	medium	spraint	actively exposed	>4	129	145	137	139	211	211	135	151	140	142	172	196	150	150	M
**Q066**	60	23.03.2010	small	mucous spraint	passively exposed	>4	129	145	135	139	211	211	135	141	140	140	176	196	150	150	M
**Q075**	5	23.03.2010	small	spraint	passively exposed	3-4	129	145	135	139	211	211	135	141	140	140	176	180	150	150	F
**Q080**	55	23.03.2010	small	spraint	passively exposed	3-4	129	145	137	137	211	211	141	141	140	142	172	196	150	150	F
**Q083**	59	23.03.2010	small	spraint	non-exposed	3-4	129	129	137	139	211	215	135	141	140	142	172	180	150	150	M
**Q093**	55	23.03.2010	small	mucous spraint	passively exposed	3-4	129	145	137	137	211	211	141	141	140	142	172	196	150	150	F
**Q100**	55	23.03.2010	small	mucous spraint	passively exposed	1-2	129	145	137	137	211	211	141	141	140	142	172	196	150	150	F
**Q112**	59	23.03.2010	small	mucous spraint	actively exposed	3-4	129	129	137	139	211	215	135	141	140	142	172	180	150	150	M
**Q113**	54	23.03.2010	small	jelly	passively exposed	3-4	145	145	137	141	211	211	135	141	140	142	180	196	150	150	F
**R003**	54	24.03.2010	medium	mucous spraint	non-exposed	3-4	145	145	137	141	211	211	135	141	140	142	180	196	150	150	F
**R004**	53	24.03.2010	medium	spraint	non-exposed	3-4	145	145	135	137	211	211	135	141	140	142	176	180	150	150	F
**R008**	63	24.03.2010	large	jelly	actively exposed	1-2	145	145	137	139	211	211	135	135	140	142	176	180	146	150	M
**R011**	53	24.03.2010	medium	spraint	actively exposed	3-4	145	145	135	137	211	211	135	141	140	142	176	180	150	150	F
**R015**	56	24.03.2010	small	jelly	passively exposed	1-2	129	145	137	139	211	211	141	151	140	142	180	196	150	150	M
**R016**	55	24.03.2010	medium	spraint	non-exposed	>4	129	145	137	137	211	211	141	141	140	142	172	196	150	150	F
**R021**	55	24.03.2010	large	spraint	passively exposed	>4	129	145	137	137	211	211	141	141	140	142	172	196	150	150	F
**R022**	55	24.03.2010	medium	spraint	actively exposed	>4	129	145	137	137	211	211	141	141	140	142	172	196	150	150	F
**R025**	55	24.03.2010	medium	spraint	actively exposed	>4	129	145	137	137	211	211	141	141	140	142	172	196	150	150	F
**R035**	44	24.03.2010	medium	jelly	non-exposed	3-4	129	129	137	139	207	211	135	141	140	142	180	180	146	150	F
**R038**	44	24.03.2010	small	spraint	passively exposed	3-4	129	129	137	139	207	211	135	141	140	142	180	180	146	150	F
**R042**	44	24.03.2010	large	spraint	non-exposed	1-2	129	129	137	139	207	211	135	141	140	142	180	180	146	150	F
**R045**	58	24.03.2010	small	mucous spraint	non-exposed	1-2	129	145	135	137	211	211	135	135	140	142	176	180	150	150	F
**R046**	84	24.03.2010	small	mucous spraint	non-exposed	1-2	129	145	135	137	211	215	135	135	140	142	176	180	150	150	F
**R048**	1	24.03.2010	small	jelly	non-exposed	1-2	129	129	135	137	211	211	135	135	140	142	172	180	150	150	F
**R049**	1	24.03.2010	large	mucous spraint	passively exposed	3-4	129	129	135	137	211	211	135	135	140	142	172	180	150	150	F

**R051**	1	24.03.2010	small	mucous spraint	passively exposed	3-4	129	129	135	137	211	211	135	135	140	142	172	180	150	150	F
**R053**	1	24.03.2010	medium	jelly	passively exposed	1-2	129	129	135	137	211	211	135	135	140	142	172	180	150	150	F
**R057**	58	24.03.2010	small	mucous spraint	non-exposed	1-2	129	145	135	137	211	211	135	135	140	142	176	180	150	150	F
**R058**	58	24.03.2010	small	jelly	passively exposed	>4	129	145	135	137	211	211	135	135	140	142	176	180	150	150	F
**R071**	5	24.03.2010	small	mucous spraint	actively exposed	3-4	129	145	135	139	211	211	135	141	140	140	176	180	150	150	F
**R082**	34	24.03.2010	medium	spraint	passively exposed	>4	129	145	137	139	211	211	135	151	140	142	172	196	150	150	M
**R086**	57	24.03.2010	medium	mucous spraint	actively exposed	3-4	129	147	139	139	211	211	135	135	140	142	172	172	146	150	F
**R088**	57	24.03.2010	small	jelly	actively exposed	1-2	129	147	139	139	211	211	135	135	140	142	172	172	146	150	F
**R089**	46	24.03.2010	small	NA	actively exposed	3-4	129	129	139	141	211	211	141	141	140	142	176	180	150	150	F
**R090**	46	24.03.2010	small	spraint	actively exposed	3-4	129	129	139	141	211	211	141	141	140	142	176	180	150	150	F
**R092**	45	24.03.2010	medium	spraint	non-exposed	1-2	129	129	139	141	211	211	135	141	140	140	176	180	150	150	F
**R096**	46	24.03.2010	small	mucous spraint	non-exposed	3-4	129	129	139	141	211	211	141	141	140	142	176	180	150	150	F
**R097**	45	24.03.2010	small	spraint	passively exposed	1-2	129	129	139	141	211	211	135	141	140	140	176	180	150	150	F
**R098**	42	24.03.2010	small	jelly	non-exposed	3-4	129	139	135	139	211	211	135	141	140	142	176	180	150	150	M
**R099**	45	24.03.2010	small	jelly	non-exposed	3-4	129	129	139	141	211	211	135	141	140	140	176	180	150	150	F
**R100**	42	24.03.2010	small	jelly	passively exposed	1-2	129	139	135	139	211	211	135	141	140	142	176	180	150	150	M
**R101**	42	24.03.2010	large	mucous spraint	non-exposed	3-4	129	139	135	139	211	211	135	141	140	142	176	180	150	150	M
**R105**	59	24.03.2010	small	jelly	passively exposed	3-4	129	129	137	139	211	215	135	141	140	142	172	180	150	150	M
**R110**	57	24.03.2010	large	mucous spraint	non-exposed	3-4	129	147	139	139	211	211	135	135	140	142	172	172	146	150	F
**R111**	57	24.03.2010	large	mucous spraint	passively exposed	3-4	129	147	139	139	211	211	135	135	140	142	172	172	146	150	F
**R112**	57	24.03.2010	large	spraint	non-exposed	3-4	129	147	139	139	211	211	135	135	140	142	172	172	146	150	F
**R114**	45	24.03.2010	small	jelly	non-exposed	3-4	129	129	139	141	211	211	135	141	140	140	176	180	150	150	F
**S001**	17	25.03.2010	small	jelly	actively exposed	1-2	145	145	137	139	211	211	135	135	140	142	176	180	146	150	F
**S004**	59	25.03.2010	small	jelly	non-exposed	>4	129	129	137	139	211	215	135	141	140	142	172	180	150	150	M
**S016**	57	25.03.2010	small	mucous spraint	non-exposed	3-4	129	147	139	139	211	211	135	135	140	142	172	172	146	150	F
**S021**	45	25.03.2010	small	mucous spraint	passively exposed	3-4	129	129	139	141	211	211	135	141	140	140	176	180	150	150	F
**S022**	46	25.03.2010	small	mucous spraint	actively exposed	3-4	129	129	139	141	211	211	141	141	140	142	176	180	150	150	F
**S024**	42	25.03.2010	small	mucous spraint	passively exposed	3-4	129	139	135	139	211	211	135	141	140	142	176	180	150	150	M
**S047**	44	25.03.2010	medium	mucous spraint	non-exposed	3-4	129	129	137	139	207	211	135	141	140	142	180	180	146	150	F
**S048**	34	25.03.2010	small	mucous spraint	non-exposed	3-4	129	145	137	139	211	211	135	151	140	142	172	196	150	150	M
**S051**	1	25.03.2010	large	mucous spraint	passively exposed	>4	129	129	135	137	211	211	135	135	140	142	172	180	150	150	F
**S053**	1	25.03.2010	medium	jelly	passively exposed	>4	129	129	135	137	211	211	135	135	140	142	172	180	150	150	F
**S059**	1	25.03.2010	medium	spraint	actively exposed	3-4	129	129	135	137	211	211	135	135	140	142	172	180	150	150	F
**S060**	58	25.03.2010	small	spraint	passively exposed	1-2	129	145	135	137	211	211	135	135	140	142	176	180	150	150	F
**S076**	56	25.03.2010	medium	mucous spraint	actively exposed	3-4	129	145	137	139	211	211	141	151	140	142	180	196	150	150	M
**S082**	56	25.03.2010	medium	spraint	actively exposed	>4	129	145	137	139	211	211	141	151	140	142	180	196	150	150	M
**S089**	34	25.03.2010	small	mucous spraint	actively exposed	>4	129	145	137	139	211	211	135	151	140	142	172	196	150	150	M
**S091**	57	25.03.2010	large	spraint	passively exposed	3-4	129	147	139	139	211	211	135	135	140	142	172	172	146	150	F
**S105**	5	25.03.2010	small	spraint	passively exposed	3-4	129	145	135	139	211	211	135	141	140	140	176	180	150	150	F
**T001**	58	26.03.2010	medium	mucous spraint	passively exposed	>4	129	145	135	137	211	211	135	135	140	142	176	180	150	150	F
**T002**	1	26.03.2010	large	jelly	passively exposed	>4	129	129	135	137	211	211	135	135	140	142	172	180	150	150	F
**T004**	1	26.03.2010	medium	jelly	passively exposed	>4	129	129	135	137	211	211	135	135	140	142	172	180	150	150	F

**T005**	1	26.03.2010	large	mucous spraint	non-exposed	>4	129	129	135	137	211	211	135	135	140	142	172	180	150	150	F
**T009**	34	26.03.2010	medium	jelly	passively exposed	3-4	129	145	137	139	211	211	135	151	140	142	172	196	150	150	M
**T010**	58	26.03.2010	medium	spraint	non-exposed	3-4	129	145	135	137	211	211	135	135	140	142	176	180	150	150	F
**T022**	44	26.03.2010	medium	mucous spraint	non-exposed	1-2	129	129	137	139	207	211	135	141	140	142	180	180	146	150	F
**T029**	46	26.03.2010	small	spraint	actively exposed	>4	129	129	139	141	211	211	141	141	140	142	176	180	150	150	F
**T037**	46	26.03.2010	medium	mucous spraint	actively exposed	>4	129	129	139	141	211	211	141	141	140	142	176	180	150	150	F
**T043**	45	26.03.2010	medium	mucous spraint	non-exposed	1-2	129	129	139	141	211	211	135	141	140	140	176	180	150	150	F
**T048**	57	26.03.2010	medium	spraint	passively exposed	>4	129	147	139	139	211	211	135	135	140	142	172	172	146	150	F
**T054**	57	26.03.2010	small	jelly	passively exposed	>4	129	147	139	139	211	211	135	135	140	142	172	172	146	150	F
**T055**	57	26.03.2010	small	jelly	passively exposed	>4	129	147	139	139	211	211	135	135	140	142	172	172	146	150	F
**T056**	55	26.03.2010	large	mucous spraint	passively exposed	3-4	129	145	137	137	211	211	141	141	140	142	172	196	150	150	F
**T057**	55	26.03.2010	large	mucous spraint	passively exposed	3-4	129	145	137	137	211	211	141	141	140	142	172	196	150	150	F
**T058**	55	26.03.2010	large	mucous spraint	actively exposed	1-2	129	145	137	137	211	211	141	141	140	142	172	196	150	150	F
**T061**	55	26.03.2010	large	spraint	actively exposed	>4	129	145	137	137	211	211	141	141	140	142	172	196	150	150	F
**T066**	55	26.03.2010	medium	spraint	non-exposed	>4	129	145	137	137	211	211	141	141	140	142	172	196	150	150	F
**T073**	59	26.03.2010	small	jelly	passively exposed	3-4	129	129	137	139	211	215	135	141	140	142	172	180	150	150	M
**T077**	55	26.03.2010	medium	spraint	actively exposed	3-4	129	145	137	137	211	211	141	141	140	142	172	196	150	150	F
**T088**	59	26.03.2010	small	mucous spraint	passively exposed	1-2	129	129	137	139	211	215	135	141	140	142	172	180	150	150	M
**T089**	17	26.03.2010	small	mucous spraint	passively exposed	1-2	145	145	137	139	211	211	135	135	140	142	176	180	146	150	F
**T091**	59	26.03.2010	medium	spraint	passively exposed	1-2	129	129	137	139	211	215	135	141	140	142	172	180	150	150	M
**T093**	59	26.03.2010	medium	spraint	passively exposed	1-2	129	129	137	139	211	215	135	141	140	142	172	180	150	150	M
**T094**	17	26.03.2010	small	mucous spraint	passively exposed	3-4	145	145	137	139	211	211	135	135	140	142	176	180	146	150	F
**T107**	57	26.03.2010	large	jelly	passively exposed	3-4	129	147	139	139	211	211	135	135	140	142	172	172	146	150	F
**V005**	65	28.03.2011	medium	spraint	passively exposed	>4	129	129	137	137	211	211	135	135	140	142	172	172	150	150	M
**V019**	53	28.03.2011	small	spraint	non-exposed	1-2	145	145	135	137	211	211	135	141	140	142	176	180	150	150	F
**V022**	21	28.03.2011	medium	mucous spraint	non-exposed	>4	145	145	135	141	211	211	135	141	140	142	176	196	150	150	M
**V032**	59	28.03.2011	small	jelly	actively exposed	1-2	129	129	137	139	211	215	135	141	140	142	172	180	150	150	M
**V046**	56	28.03.2011	medium	jelly	non-exposed	1-2	129	145	137	139	211	211	141	151	140	142	180	196	150	150	M
**V050**	21	28.03.2011	medium	spraint	actively exposed	1-2	145	145	135	141	211	211	135	141	140	142	176	196	150	150	M
**V052**	44	28.03.2011	medium	spraint	non-exposed	1-2	129	129	137	139	207	211	135	141	140	142	180	180	146	150	F
**V055**	71	28.03.2011	large	jelly	non-exposed	1-2	129	147	135	139	211	211	135	135	140	140	172	180	146	150	F
**V064**	73	28.03.2011	large	spraint	non-exposed	>4	129	139	139	139	211	211	135	141	140	142	176	180	150	150	M
**V068**	65	28.03.2011	medium	mucous spraint	non-exposed	1-2	129	129	137	137	211	211	135	135	140	142	172	172	150	150	M
**V074**	58	28.03.2011	medium	mucous spraint	non-exposed	1-2	129	145	135	137	211	211	135	135	140	142	176	180	150	150	F
**V085**	66	28.03.2011	medium	spraint	passively exposed	3-4	129	129	135	141	211	211	141	141	140	142	180	180	150	150	F
**V087**	73	28.03.2011	medium	mucous spraint	non-exposed	3-4	129	139	139	139	211	211	135	141	140	142	176	180	150	150	M
**V097**	42	28.03.2011	small	mucous spraint	non-exposed	3-4	129	139	135	139	211	211	135	141	140	142	176	180	150	150	M
**V101**	57	28.03.2011	small	spraint	non-exposed	3-4	129	147	139	139	211	211	135	135	140	142	172	172	146	150	F
**V104**	67	28.03.2011	large	spraint	non-exposed	3-4	129	129	137	141	211	211	135	151	140	142	180	196	146	150	M
**V109**	67	28.03.2011	small	mucous spraint	passively exposed	1-2	129	129	137	141	211	211	135	151	140	142	180	196	146	150	M
**V116**	59	28.03.2011	medium	mucous spraint	actively exposed	3-4	129	129	137	139	211	215	135	141	140	142	172	180	150	150	M
**V118**	44	28.03.2011	medium	spraint	non-exposed	3-4	129	129	137	139	207	211	135	141	140	142	180	180	146	150	F

**V127**	69	28.03.2011	small	spraint	actively exposed	>4	129	145	139	139	211	211	135	141	140	140	172	176	146	150	F
**W002**	71	29.03.2011	large	spraint	passively exposed	3-4	129	147	135	139	211	211	135	135	140	140	172	180	146	150	F
**W004**	42	29.03.2011	medium	spraint	passively exposed	1-2	129	139	135	139	211	211	135	141	140	142	176	180	150	150	M
**W005**	59	29.03.2011	large	mucous spraint	actively exposed	>4	129	129	137	139	211	215	135	141	140	142	172	180	150	150	M
**W006**	71	29.03.2011	large	mucous spraint	passively exposed	3-4	129	147	135	139	211	211	135	135	140	140	172	180	146	150	F
**W011**	55	29.03.2011	large	jelly	non-exposed	3-4	129	145	137	137	211	211	141	141	140	142	172	196	150	150	F
**W013**	57	29.03.2011	large	jelly	passively exposed	3-4	129	147	139	139	211	211	135	135	140	142	172	172	146	150	F
**W020**	69	29.03.2011	small	spraint	passively exposed	1-2	129	145	139	139	211	211	135	141	140	140	172	176	146	150	F
**W026**	69	29.03.2011	small	spraint	passively exposed	3-4	129	145	139	139	211	211	135	141	140	140	172	176	146	150	F
**W030**	66	29.03.2011	medium	mucous spraint	non-exposed	>4	129	129	135	141	211	211	141	141	140	142	180	180	150	150	F
**W034**	17	29.03.2011	large	spraint	non-exposed	1-2	145	145	137	139	211	211	135	135	140	142	176	180	146	150	F
**W040**	68	29.03.2011	large	mucous spraint	non-exposed	>4	129	139	135	141	211	211	135	141	140	142	176	180	150	150	M
**W042**	59	29.03.2011	large	mucous spraint	non-exposed	3-4	129	129	137	139	211	215	135	141	140	142	172	180	150	150	M
**W049**	68	29.03.2011	medium	spraint	non-exposed	>4	129	139	135	141	211	211	135	141	140	142	176	180	150	150	M
**W050**	21	29.03.2011	large	mucous spraint	actively exposed	>4	145	145	135	141	211	211	135	141	140	142	176	196	150	150	M
**W053**	68	29.03.2011	medium	spraint	passively exposed	>4	129	139	135	141	211	211	135	141	140	142	176	180	150	150	M
**W055**	68	29.03.2011	small	mucous spraint	non-exposed	>4	129	139	135	141	211	211	135	141	140	142	176	180	150	150	M
**W061**	68	29.03.2011	large	mucous spraint	passively exposed	>4	129	139	135	141	211	211	135	141	140	142	176	180	150	150	M
**W065**	44	29.03.2011	small	mucous spraint	non-exposed	1-2	129	129	137	139	207	211	135	141	140	142	180	180	146	150	F
**W076**	68	29.03.2011	large	spraint	non-exposed	>4	129	139	135	141	211	211	135	141	140	142	176	180	150	150	M
**W084**	57	29.03.2011	large	spraint	actively exposed	1-2	129	147	139	139	211	211	135	135	140	142	172	172	146	150	F
**W093**	59	29.03.2011	large	jelly	passively exposed	>4	129	129	137	139	211	215	135	141	140	142	172	180	150	150	M
**W097**	59	29.03.2011	large	jelly	non-exposed	3-4	129	129	137	139	211	215	135	141	140	142	172	180	150	150	M
**W098**	44	29.03.2011	large	mucous spraint	actively exposed	3-4	129	129	137	139	207	211	135	141	140	142	180	180	146	150	F
**W108**	74	29.03.2011	medium	mucous spraint	passively exposed	1-2	129	145	137	139	211	211	135	141	140	142	172	176	150	150	M
**W112**	44	29.03.2011	medium	jelly	actively exposed	3-4	129	129	137	139	207	211	135	141	140	142	180	180	146	150	F
**W114**	72	29.03.2011	large	spraint	non-exposed	3-4	139	147	139	139	211	211	135	135	140	140	172	176	150	150	F
**W117**	74	29.03.2011	small	mucous spraint	passively exposed	1-2	129	145	137	139	211	211	135	141	140	142	172	176	150	150	M
**W118**	58	29.03.2011	medium	jelly	passively exposed	>4	129	145	135	137	211	211	135	135	140	142	176	180	150	150	F
**X005**	44	30.03.2011	large	spraint	actively exposed	3-4	129	129	137	139	207	211	135	141	140	142	180	180	146	150	F
**X008**	59	30.03.2011	small	mucous spraint	actively exposed	>4	129	129	137	139	211	215	135	141	140	142	172	180	150	150	M
**X018**	58	30.03.2011	small	spraint	non-exposed	3-4	129	145	135	137	211	211	135	135	140	142	176	180	150	150	F
**X031**	76	30.03.2011	medium	spraint	non-exposed	>4	129	145	135	137	211	211	135	135	140	142	176	176	150	150	F
**X040**	59	30.03.2011	large	jelly	actively exposed	>4	129	129	137	139	211	215	135	141	140	142	172	180	150	150	M
**X041**	44	30.03.2011	small	spraint	passively exposed	3-4	129	129	137	139	207	211	135	141	140	142	180	180	146	150	F
**X042**	44	30.03.2011	medium	spraint	non-exposed	3-4	129	129	137	139	207	211	135	141	140	142	180	180	146	150	F
**X046**	64	30.03.2011	medium	mucous spraint	non-exposed	>4	129	129	135	139	211	211	135	135	140	142	172	196	150	150	M
**X050**	64	30.03.2011	small	jelly	non-exposed	>4	129	129	135	139	211	211	135	135	140	142	172	196	150	150	M
**X051**	58	30.03.2011	medium	spraint	non-exposed	3-4	129	145	135	137	211	211	135	135	140	142	176	180	150	150	F
**X054**	69	30.03.2011	small	mucous spraint	passively exposed	3-4	129	145	139	139	211	211	135	141	140	140	172	176	146	150	F
**X058**	21	30.03.2011	small	mucous spraint	actively exposed	3-4	145	145	135	141	211	211	135	141	140	142	176	196	150	150	M
**X059**	21	30.03.2011	medium	mucous spraint	actively exposed	3-4	145	145	135	141	211	211	135	141	140	142	176	196	150	150	M

**X063**	70	30.03.2011	large	spraint	actively exposed	3-4	129	145	139	141	211	211	135	141	140	142	180	180	150	150	F
**X067**	70	30.03.2011	medium	mucous spraint	actively exposed	1-2	129	145	139	141	211	211	135	141	140	142	180	180	150	150	F
**X068**	21	30.03.2011	small	mucous spraint	actively exposed	3-4	145	145	135	141	211	211	135	141	140	142	176	196	150	150	M
**X070**	21	30.03.2011	small	mucous spraint	actively exposed	1-2	145	145	135	141	211	211	135	141	140	142	176	196	150	150	M
**X072**	21	30.03.2011	small	mucous spraint	actively exposed	3-4	145	145	135	141	211	211	135	141	140	142	176	196	150	150	M
**X074**	70	30.03.2011	medium	mucous spraint	actively exposed	1-2	129	145	139	141	211	211	135	141	140	142	180	180	150	150	F
**X077**	70	30.03.2011	small	jelly	actively exposed	3-4	129	145	139	141	211	211	135	141	140	142	180	180	150	150	F
**X079**	21	30.03.2011	small	mucous spraint	actively exposed	3-4	145	145	135	141	211	211	135	141	140	142	176	196	150	150	M
**X100**	46	30.03.2011	medium	spraint	actively exposed	>4	129	129	139	141	211	211	141	141	140	142	176	180	150	150	F
**X104**	46	30.03.2011	medium	spraint	actively exposed	>4	129	129	139	141	211	211	141	141	140	142	176	180	150	150	F
**X107**	73	30.03.2011	large	mucous spraint	non-exposed	>4	129	139	139	139	211	211	135	141	140	142	176	180	150	150	M
**X116**	46	30.03.2011	large	spraint	actively exposed	1-2	129	129	139	141	211	211	141	141	140	142	176	180	150	150	F
**X117**	46	30.03.2011	medium	spraint	actively exposed	3-4	129	129	139	141	211	211	141	141	140	142	176	180	150	150	F
**X118**	46	30.03.2011	large	spraint	non-exposed	3-4	129	129	139	141	211	211	141	141	140	142	176	180	150	150	F
**X120**	46	30.03.2011	large	spraint	actively exposed	3-4	129	129	139	141	211	211	141	141	140	142	176	180	150	150	F
**X121**	68	30.03.2011	large	mucous spraint	non-exposed	>4	129	139	135	141	211	211	135	141	140	142	176	180	150	150	M
**X124**	68	30.03.2011	large	spraint	non-exposed	>4	129	139	135	141	211	211	135	141	140	142	176	180	150	150	M
**X131**	46	30.03.2011	large	spraint	actively exposed	1-2	129	129	139	141	211	211	141	141	140	142	176	180	150	150	F
**X137**	21	30.03.2011	medium	spraint	actively exposed	3-4	145	145	135	141	211	211	135	141	140	142	176	196	150	150	M
**X139**	57	30.03.2011	large	spraint	non-exposed	3-4	129	147	139	139	211	211	135	135	140	142	172	172	146	150	F
**X152**	53	30.03.2011	medium	spraint	non-exposed	3-4	145	145	135	137	211	211	135	141	140	142	176	180	150	150	F
**X154**	71	30.03.2011	medium	mucous spraint	non-exposed	3-4	129	147	135	139	211	211	135	135	140	140	172	180	146	150	F
**X155**	54	30.03.2011	large	mucous spraint	passively exposed	1-2	145	145	137	141	211	211	135	141	140	142	180	196	150	150	F
**XA001**	81	27.03.2012	small	spraint	actively exposed	>4	129	139	135	135	203	211	135	141	140	142	176	180	150	150	M
**XA019**	59	27.03.2012	large	spraint	actively exposed	3-4	129	129	137	139	211	215	135	141	140	142	172	180	150	150	M
**XA020**	79	27.03.2012	medium	spraint	actively exposed	3-4	129	139	135	137	211	211	135	141	142	148	176	176	150	150	M
**XA056**	69	27.03.2012	large	mucous spraint	non-exposed	>4	129	145	139	139	211	211	135	141	140	140	172	176	146	150	F
**XA058**	81	27.03.2012	small	spraint	actively exposed	>4	129	139	135	135	203	211	135	141	140	142	176	180	150	150	M
**XA064**	69	27.03.2012	small	jelly	actively exposed	>4	129	145	139	139	211	211	135	141	140	140	172	176	146	150	F
**XA074**	78	27.03.2012	medium	jelly	non-exposed	3-4	129	129	137	137	211	211	141	141	140	142	180	196	150	150	F
**XA088**	46	27.03.2012	small	jelly	actively exposed	1-2	129	129	139	141	211	211	141	141	140	142	176	180	150	150	F
**XA090**	71	27.03.2012	small	jelly	actively exposed	1-2	129	147	135	139	211	211	135	135	140	140	172	180	146	150	F
**XA099**	59	27.03.2012	large	spraint	actively exposed	3-4	129	129	137	139	211	215	135	141	140	142	172	180	150	150	M
**XA103**	79	27.03.2012	large	mucous spraint	passively exposed	3-4	129	139	135	137	211	211	135	141	142	148	176	176	150	150	M
**XA104**	46	27.03.2012	small	jelly	actively exposed	1-2	129	129	139	141	211	211	141	141	140	142	176	180	150	150	F
**XA108**	44	27.03.2012	medium	mucous spraint	passively exposed	3-4	129	129	137	139	207	211	135	141	140	142	180	180	146	150	F
**XA115**	69	27.03.2012	small	mucous spraint	actively exposed	3-4	129	145	139	139	211	211	135	141	140	140	172	176	146	150	F
**XA117**	67	27.03.2012	large	jelly	passively exposed	3-4	129	129	137	141	211	211	135	151	140	142	180	196	146	150	M
**XA126**	59	27.03.2012	large	spraint	passively exposed	3-4	129	129	137	139	211	215	135	141	140	142	172	180	150	150	M
**XB002**	81	28.03.2012	medium	spraint	actively exposed	1-2	129	139	135	135	203	211	135	141	140	142	176	180	150	150	M
**XB004**	73	28.03.2012	large	spraint	actively exposed	>4	129	139	139	139	211	211	135	141	140	142	176	180	150	150	M
**XB005**	81	28.03.2012	medium	mucous spraint	passively exposed	>4	129	139	135	135	203	211	135	141	140	142	176	180	150	150	M

**XB007**	66	28.03.2012	small	jelly	non-exposed	>4	129	129	135	141	211	211	141	141	140	142	180	180	150	150	F
**XB008**	53	28.03.2012	small	jelly	actively exposed	1-2	145	145	135	137	211	211	135	141	140	142	176	180	150	150	F
**XB013**	81	28.03.2012	large	mucous spraint	passively exposed	>4	129	139	135	135	203	211	135	141	140	142	176	180	150	150	M
**XB020**	71	28.03.2012	large	jelly	actively exposed	3-4	129	147	135	139	211	211	135	135	140	140	172	180	146	150	F
**XB028**	78	28.03.2012	medium	jelly	passively exposed	>4	129	129	137	137	211	211	141	141	140	142	180	196	150	150	F
**XB038**	59	28.03.2012	medium	spraint	actively exposed	3-4	129	129	137	139	211	215	135	141	140	142	172	180	150	150	M
**XB043**	77	28.03.2012	large	mucous spraint	passively exposed	>4	129	129	137	139	211	211	141	151	140	142	172	180	150	150	M
**XB046**	78	28.03.2012	large	spraint	actively exposed	1-2	129	129	137	137	211	211	141	141	140	142	180	196	150	150	F
**XB047**	78	28.03.2012	medium	spraint	non-exposed	>4	129	129	137	137	211	211	141	141	140	142	180	196	150	150	F
**XB051**	78	28.03.2012	medium	jelly	non-exposed	>4	129	129	137	137	211	211	141	141	140	142	180	196	150	150	F
**XB066**	54	28.03.2012	medium	jelly	passively exposed	>4	145	145	137	141	211	211	135	141	140	142	180	196	150	150	F
**XB075**	44	28.03.2012	large	mucous spraint	passively exposed	>4	129	129	137	139	207	211	135	141	140	142	180	180	146	150	F
**XB077**	44	28.03.2012	small	spraint	non-exposed	3-4	129	129	137	139	207	211	135	141	140	142	180	180	146	150	F
**XB081**	40	28.03.2012	medium	mucous spraint	passively exposed	>4	129	145	135	139	203	211	135	141	140	142	176	180	146	150	M
**XB084**	54	28.03.2012	small	jelly	passively exposed	>4	145	145	137	141	211	211	135	141	140	142	180	196	150	150	F
**XB089**	79	28.03.2012	small	spraint	actively exposed	>4	129	139	135	137	211	211	135	141	142	148	176	176	150	150	M
**XB116**	66	28.03.2012	medium	jelly	non-exposed	3-4	129	129	135	141	211	211	141	141	140	142	180	180	150	150	F
**XB120**	72	28.03.2012	large	mucous spraint	actively exposed	1-2	139	147	139	139	211	211	135	135	140	140	172	176	150	150	F
**XB121**	35	28.03.2012	medium	mucous spraint	actively exposed	>4	129	129	137	139	207	211	141	141	140	142	180	180	146	150	F
**XB122**	72	28.03.2012	medium	mucous spraint	actively exposed	>4	139	147	139	139	211	211	135	135	140	140	172	176	150	150	F
**XB129**	46	28.03.2012	small	mucous spraint	actively exposed	1-2	129	129	139	141	211	211	141	141	140	142	176	180	150	150	F
**XB132**	72	28.03.2012	small	mucous spraint	actively exposed	>4	139	147	139	139	211	211	135	135	140	140	172	176	150	150	F
**XB133**	71	28.03.2012	medium	mucous spraint	actively exposed	>4	129	147	135	139	211	211	135	135	140	140	172	180	146	150	F
**XB138**	72	28.03.2012	large	mucous spraint	actively exposed	>4	139	147	139	139	211	211	135	135	140	140	172	176	150	150	F
**XB142**	57	28.03.2012	small	mucous spraint	actively exposed	>4	129	147	139	139	211	211	135	135	140	142	172	172	146	150	F
**XB146**	68	28.03.2012	small	mucous spraint	passively exposed	1-2	129	139	135	141	211	211	135	141	140	142	176	180	150	150	M
**XB147**	71	28.03.2012	large	spraint	actively exposed	>4	129	147	135	139	211	211	135	135	140	140	172	180	146	150	F
**XC008**	71	29.03.2012	small	jelly	actively exposed	>4	129	147	135	139	211	211	135	135	140	140	172	180	146	150	F
**XC012**	71	29.03.2012	large	spraint	actively exposed	3-4	129	147	135	139	211	211	135	135	140	140	172	180	146	150	F
**XC013**	73	29.03.2012	medium	jelly	actively exposed	>4	129	139	139	139	211	211	135	141	140	142	176	180	150	150	M
**XC015**	71	29.03.2012	medium	mucous spraint	actively exposed	>4	129	147	135	139	211	211	135	135	140	140	172	180	146	150	F
**XC021**	72	29.03.2012	medium	spraint	actively exposed	3-4	139	147	139	139	211	211	135	135	140	140	172	176	150	150	F
**XC036**	81	29.03.2012	large	spraint	actively exposed	>4	129	139	135	135	203	211	135	141	140	142	176	180	150	150	M
**XC037**	35	29.03.2012	large	spraint	actively exposed	>4	129	129	137	139	207	211	141	141	140	142	180	180	146	150	F
**XC040**	72	29.03.2012	medium	mucous spraint	actively exposed	>4	139	147	139	139	211	211	135	135	140	140	172	176	150	150	F
**XC042**	44	29.03.2012	medium	mucous spraint	non-exposed	3-4	129	129	137	139	207	211	135	141	140	142	180	180	146	150	F
**XC046**	82	29.03.2012	large	spraint	non-exposed	1-2	129	129	137	137	211	211	141	141	140	142	180	180	146	150	F
**XC047**	35	29.03.2012	medium	mucous spraint	actively exposed	>4	129	129	137	139	207	211	141	141	140	142	180	180	146	150	F
**XC050**	44	29.03.2012	large	spraint	actively exposed	3-4	129	129	137	139	207	211	135	141	140	142	180	180	146	150	F
**XC052**	44	29.03.2012	small	spraint	actively exposed	>4	129	129	137	139	207	211	135	141	140	142	180	180	146	150	F
**XC053**	35	29.03.2012	medium	mucous spraint	actively exposed	>4	129	129	137	139	207	211	141	141	140	142	180	180	146	150	F
**XC054**	72	29.03.2012	small	jelly	actively exposed	3-4	139	147	139	139	211	211	135	135	140	140	172	176	150	150	F

**XC055**	71	29.03.2012	medium	spraint	actively exposed	>4	129	147	135	139	211	211	135	135	140	140	172	180	146	150	F
**XC057**	72	29.03.2012	medium	mucous spraint	actively exposed	3-4	139	147	139	139	211	211	135	135	140	140	172	176	150	150	F
**XC058**	72	29.03.2012	large	spraint	actively exposed	>4	139	147	139	139	211	211	135	135	140	140	172	176	150	150	F
**XC063**	67	29.03.2012	medium	spraint	non-exposed	3-4	129	129	137	141	211	211	135	151	140	142	180	196	146	150	M
**XC070**	67	29.03.2012	medium	spraint	passively exposed	3-4	129	129	137	141	211	211	135	151	140	142	180	196	146	150	M
**XC073**	82	29.03.2012	small	mucous spraint	non-exposed	3-4	129	129	137	137	211	211	141	141	140	142	180	180	146	150	F
**XC100**	55	29.03.2012	large	spraint	actively exposed	1-2	129	145	137	137	211	211	141	141	140	142	172	196	150	150	F
**XC115**	81	29.03.2012	small	spraint	actively exposed	>4	129	139	135	135	203	211	135	141	140	142	176	180	150	150	M
**XC116**	68	29.03.2012	large	mucous spraint	actively exposed	>4	129	139	135	141	211	211	135	141	140	142	176	180	150	150	M
**XC117**	68	29.03.2012	large	mucous spraint	actively exposed	>4	129	139	135	141	211	211	135	141	140	142	176	180	150	150	M
**XC118**	68	29.03.2012	small	mucous spraint	actively exposed	3-4	129	139	135	141	211	211	135	141	140	142	176	180	150	150	M
**XC119**	69	29.03.2012	large	spraint	passively exposed	1-2	129	145	139	139	211	211	135	141	140	140	172	176	146	150	F
**XC126**	69	29.03.2012	large	mucous spraint	passively exposed	1-2	129	145	139	139	211	211	135	141	140	140	172	176	146	150	F
**XC127**	81	29.03.2012	medium	mucous spraint	passively exposed	1-2	129	139	135	135	203	211	135	141	140	142	176	180	150	150	M
**XC131**	66	29.03.2012	medium	mucous spraint	actively exposed	>4	129	129	135	141	211	211	141	141	140	142	180	180	150	150	F
**XC134**	73	29.03.2012	small	jelly	actively exposed	3-4	129	139	139	139	211	211	135	141	140	142	176	180	150	150	M
**XC138**	68	29.03.2012	large	mucous spraint	actively exposed	>4	129	139	135	141	211	211	135	141	140	142	176	180	150	150	M
**XC148**	73	29.03.2012	small	jelly	actively exposed	1-2	129	139	139	139	211	211	135	141	140	142	176	180	150	150	M
**XC150**	66	29.03.2012	large	NA	actively exposed	>4	129	129	135	141	211	211	141	141	140	142	180	180	150	150	F
**XD002**	81	30.03.2012	medium	jelly	actively exposed	3-4	129	139	135	135	203	211	135	141	140	142	176	180	150	150	M
**XD007**	72	30.03.2012	large	jelly	non-exposed	3-4	139	147	139	139	211	211	135	135	140	140	172	176	150	150	F
**XD008**	71	30.03.2012	large	spraint	actively exposed	>4	129	147	135	139	211	211	135	135	140	140	172	180	146	150	F
**XD009**	73	30.03.2012	large	mucous spraint	actively exposed	3-4	129	139	139	139	211	211	135	141	140	142	176	180	150	150	M
**XD010**	54	30.03.2012	large	spraint	passively exposed	>4	145	145	137	141	211	211	135	141	140	142	180	196	150	150	F
**XD022**	81	30.03.2012	medium	jelly	passively exposed	3-4	129	139	135	135	203	211	135	141	140	142	176	180	150	150	M
**XD025**	63	30.03.2012	small	spraint	passively exposed	1-2	145	145	137	139	211	211	135	135	140	142	176	180	146	150	M
**XD027**	57	30.03.2012	large	mucous spraint	actively exposed	>4	129	147	139	139	211	211	135	135	140	142	172	172	146	150	F
**XD028**	44	30.03.2012	medium	spraint	non-exposed	>4	129	129	137	139	207	211	135	141	140	142	180	180	146	150	F
**XD029**	68	30.03.2012	medium	mucous spraint	actively exposed	>4	129	139	135	141	211	211	135	141	140	142	176	180	150	150	M
**XD035**	81	30.03.2012	medium	jelly	actively exposed	3-4	129	139	135	135	203	211	135	141	140	142	176	180	150	150	M
**XD040**	57	30.03.2012	large	spraint	actively exposed	>4	129	147	139	139	211	211	135	135	140	142	172	172	146	150	F
**XD043**	66	30.03.2012	large	mucous spraint	actively exposed	>4	129	129	135	141	211	211	141	141	140	142	180	180	150	150	F
**XD044**	81	30.03.2012	small	jelly	actively exposed	>4	129	139	135	135	203	211	135	141	140	142	176	180	150	150	M
**XD046**	68	30.03.2012	large	jelly	actively exposed	>4	129	139	135	141	211	211	135	141	140	142	176	180	150	150	M
**XD048**	71	30.03.2012	large	spraint	actively exposed	>4	129	147	135	139	211	211	135	135	140	140	172	180	146	150	F
**XD049**	68	30.03.2012	medium	jelly	actively exposed	>4	129	139	135	141	211	211	135	141	140	142	176	180	150	150	M
**XD053**	81	30.03.2012	small	jelly	passively exposed	>4	129	139	135	135	203	211	135	141	140	142	176	180	150	150	M
**XD056**	44	30.03.2012	medium	mucous spraint	actively exposed	>4	129	129	137	139	207	211	135	141	140	142	180	180	146	150	F
**XD057**	68	30.03.2012	medium	jelly	actively exposed	3-4	129	139	135	141	211	211	135	141	140	142	176	180	150	150	M
**XD062**	35	30.03.2012	medium	mucous spraint	actively exposed	3-4	129	129	137	139	207	211	141	141	140	142	180	180	146	150	F
**XD067**	73	30.03.2012	large	jelly	actively exposed	3-4	129	139	139	139	211	211	135	141	140	142	176	180	150	150	M
**XD078**	79	30.03.2012	small	jelly	actively exposed	3-4	129	139	135	137	211	211	135	141	142	148	176	176	150	150	M

**XD081**	78	30.03.2012	large	spraint	passively exposed	3-4	129	129	137	137	211	211	141	141	140	142	180	196	150	150	F
**XD086**	78	30.03.2012	large	mucous spraint	passively exposed	>4	129	129	137	137	211	211	141	141	140	142	180	196	150	150	F
**XD090**	59	30.03.2012	large	spraint	actively exposed	3-4	129	129	137	139	211	215	135	141	140	142	172	180	150	150	M
**XD095**	46	30.03.2012	small	jelly	actively exposed	3-4	129	129	139	141	211	211	141	141	140	142	176	180	150	150	F
**XD096**	73	30.03.2012	medium	jelly	actively exposed	1-2	129	139	139	139	211	211	135	141	140	142	176	180	150	150	M
**XD097**	68	30.03.2012	medium	spraint	actively exposed	1-2	129	139	135	141	211	211	135	141	140	142	176	180	150	150	M
**XD109**	69	30.03.2012	large	spraint	non-exposed	3-4	129	145	139	139	211	211	135	141	140	140	172	176	146	150	F
**XD110**	69	30.03.2012	small	mucous spraint	non-exposed	1-2	129	145	139	139	211	211	135	141	140	140	172	176	146	150	F
**XD119**	44	30.03.2012	small	mucous spraint	actively exposed	3-4	129	129	137	139	207	211	135	141	140	142	180	180	146	150	F
**XD123**	59	30.03.2012	small	jelly	actively exposed	>4	129	129	137	139	211	215	135	141	140	142	172	180	150	150	M
**XD124**	57	30.03.2012	medium	jelly	actively exposed	>4	129	147	139	139	211	211	135	135	140	142	172	172	146	150	F
**XD125**	44	30.03.2012	small	mucous spraint	actively exposed	1-2	129	129	137	139	207	211	135	141	140	142	180	180	146	150	F
**XD129**	35	30.03.2012	small	mucous spraint	actively exposed	>4	129	129	137	139	207	211	141	141	140	142	180	180	146	150	F
**XD133**	82	30.03.2012	medium	spraint	non-exposed	1-2	129	129	137	137	211	211	141	141	140	142	180	180	146	150	F
**XD135**	68	30.03.2012	large	jelly	actively exposed	>4	129	139	135	141	211	211	135	141	140	142	176	180	150	150	M
**XD137**	67	30.03.2012	medium	jelly	actively exposed	3-4	129	129	137	141	211	211	135	151	140	142	180	196	146	150	M
**XD138**	44	30.03.2012	medium	spraint	non-exposed	3-4	129	129	137	139	207	211	135	141	140	142	180	180	146	150	F
**XD142**	80	30.03.2012	small	mucous spraint	actively exposed	>4	129	129	137	141	211	211	135	141	142	148	176	176	150	150	F
**XD143**	59	30.03.2012	medium	jelly	actively exposed	>4	129	129	137	139	211	215	135	141	140	142	172	180	150	150	M
**XD149**	35	30.03.2012	large	jelly	non-exposed	>4	129	129	137	139	207	211	141	141	140	142	180	180	146	150	F
**XD250**	35	30.03.2012	medium	jelly	actively exposed	>4	129	129	137	139	207	211	141	141	140	142	180	180	146	150	F
**XE019**	82	31.03.2012	medium	mucous spraint	non-exposed	3-4	129	129	137	137	211	211	141	141	140	142	180	180	146	150	F
**XE031**	72	31.03.2012	large	jelly	actively exposed	>4	139	147	139	139	211	211	135	135	140	140	172	176	150	150	F
**XE034**	35	31.03.2012	large	mucous spraint	actively exposed	>4	129	129	137	139	207	211	141	141	140	142	180	180	146	150	F
**XE035**	44	31.03.2012	small	mucous spraint	passively exposed	>4	129	129	137	139	207	211	135	141	140	142	180	180	146	150	F
**XE037**	71	31.03.2012	large	jelly	actively exposed	>4	129	147	135	139	211	211	135	135	140	140	172	180	146	150	F
**XE040**	67	31.03.2012	large	mucous spraint	passively exposed	>4	129	129	137	141	211	211	135	151	140	142	180	196	146	150	M
**XE042**	72	31.03.2012	medium	spraint	actively exposed	>4	139	147	139	139	211	211	135	135	140	140	172	176	150	150	F
**XE043**	71	31.03.2012	large	jelly	actively exposed	>4	129	147	135	139	211	211	135	135	140	140	172	180	146	150	F
**XE046**	35	31.03.2012	large	mucous spraint	passively exposed	>4	129	129	137	139	207	211	141	141	140	142	180	180	146	150	F
**XE050**	35	31.03.2012	large	mucous spraint	actively exposed	>4	129	129	137	139	207	211	141	141	140	142	180	180	146	150	F
**XE060**	72	31.03.2012	large	mucous spraint	actively exposed	>4	139	147	139	139	211	211	135	135	140	140	172	176	150	150	F
**XE062**	71	31.03.2012	small	jelly	actively exposed	>4	129	147	135	139	211	211	135	135	140	140	172	180	146	150	F
**XE079**	54	31.03.2012	medium	jelly	passively exposed	3-4	145	145	137	141	211	211	135	141	140	142	180	196	150	150	F
**XE097**	59	31.03.2012	large	mucous spraint	non-exposed	3-4	129	129	137	139	211	215	135	141	140	142	172	180	150	150	M
**XE100**	59	31.03.2012	medium	mucous spraint	passively exposed	>4	129	129	137	139	211	215	135	141	140	142	172	180	150	150	M
**XE109**	59	31.03.2012	large	spraint	non-exposed	3-4	129	129	137	139	211	215	135	141	140	142	172	180	150	150	M
**XE110**	78	31.03.2012	small	mucous spraint	passively exposed	1-2	129	129	137	137	211	211	141	141	140	142	180	196	150	150	F
**XE112**	69	31.03.2012	small	mucous spraint	non-exposed	3-4	129	145	139	139	211	211	135	141	140	140	172	176	146	150	F
**XE118**	59	31.03.2012	large	spraint	passively exposed	>4	129	129	137	139	211	215	135	141	140	142	172	180	150	150	M
**XE120**	69	31.03.2012	small	jelly	passively exposed	>4	129	145	139	139	211	211	135	141	140	140	172	176	146	150	F
**XE136**	69	31.03.2012	medium	spraint	actively exposed	1-2	129	145	139	139	211	211	135	141	140	140	172	176	146	150	F

**XE151**	44	31.03.2012	large	mucous spraint	passively exposed	3-4	129	129	137	139	207	211	135	141	140	142	180	180	146	150	F
**XE152**	35	31.03.2012	large	jelly	passively exposed	>4	129	129	137	139	207	211	141	141	140	142	180	180	146	150	F
**XE155**	44	31.03.2012	medium	mucous spraint	non-exposed	>4	129	129	137	139	207	211	135	141	140	142	180	180	146	150	F
**XE156**	73	31.03.2012	medium	jelly	actively exposed	>4	129	139	139	139	211	211	135	141	140	142	176	180	150	150	M
**XE157**	49	31.03.2012	small	spraint	actively exposed	>4	129	145	139	139	207	211	135	141	140	140	180	180	150	150	F
**XE158**	80	31.03.2012	large	spraint	actively exposed	>4	129	129	137	141	211	211	135	141	142	148	176	176	150	150	F
**XE160**	46	31.03.2012	medium	spraint	actively exposed	>4	129	129	139	141	211	211	141	141	140	142	176	180	150	150	F
**XE162**	73	31.03.2012	medium	mucous spraint	actively exposed	>4	129	139	139	139	211	211	135	141	140	142	176	180	150	150	M
**XE165**	59	31.03.2012	small	jelly	passively exposed	>4	129	129	137	139	211	215	135	141	140	142	172	180	150	150	M
**XE166**	66	31.03.2012	medium	mucous spraint	actively exposed	>4	129	129	135	141	211	211	141	141	140	142	180	180	150	150	F
**XE167**	73	31.03.2012	large	mucous spraint	actively exposed	>4	129	139	139	139	211	211	135	141	140	142	176	180	150	150	M
**XE168**	72	31.03.2012	medium	spraint	actively exposed	>4	139	147	139	139	211	211	135	135	140	140	172	176	150	150	F
**XE171**	73	31.03.2012	small	spraint	actively exposed	3-4	129	139	139	139	211	211	135	141	140	142	176	180	150	150	M
**XE300**	44	31.03.2012	medium	mucous spraint	passively exposed	3-4	129	129	137	139	207	211	135	141	140	142	180	180	146	150	F
**Y004**	59	31.03.2011	small	jelly	passively exposed	>4	129	129	137	139	211	215	135	141	140	142	172	180	150	150	M
**Y008**	59	31.03.2011	small	mucous spraint	passively exposed	3-4	129	129	137	139	211	215	135	141	140	142	172	180	150	150	M
**Y012**	54	31.03.2011	medium	mucous spraint	passively exposed	3-4	145	145	137	141	211	211	135	141	140	142	180	196	150	150	F
**Y026**	42	31.03.2011	medium	spraint	non-exposed	>4	129	139	135	139	211	211	135	141	140	142	176	180	150	150	M
**Y029**	72	31.03.2011	medium	spraint	non-exposed	3-4	139	147	139	139	211	211	135	135	140	140	172	176	150	150	F
**Y031**	71	31.03.2011	medium	spraint	non-exposed	3-4	129	147	135	139	211	211	135	135	140	140	172	180	146	150	F
**Y033**	42	31.03.2011	medium	NA	actively exposed	3-4	129	139	135	139	211	211	135	141	140	142	176	180	150	150	M
**Y034**	57	31.03.2011	medium	spraint	actively exposed	3-4	129	147	139	139	211	211	135	135	140	142	172	172	146	150	F
**Y055**	42	31.03.2011	small	spraint	non-exposed	>4	129	139	135	139	211	211	135	141	140	142	176	180	150	150	M
**Y059**	66	31.03.2011	medium	spraint	passively exposed	>4	129	129	135	141	211	211	141	141	140	142	180	180	150	150	F
**Y061**	42	31.03.2011	small	spraint	actively exposed	1-2	129	139	135	139	211	211	135	141	140	142	176	180	150	150	M
**Y062**	42	31.03.2011	small	spraint	passively exposed	>4	129	139	135	139	211	211	135	141	140	142	176	180	150	150	M
**Y065**	68	31.03.2011	small	spraint	passively exposed	>4	129	139	135	141	211	211	135	141	140	142	176	180	150	150	M
**Y067**	46	31.03.2011	large	spraint	non-exposed	>4	129	129	139	141	211	211	141	141	140	142	176	180	150	150	F
**Y071**	42	31.03.2011	large	spraint	passively exposed	>4	129	139	135	139	211	211	135	141	140	142	176	180	150	150	M
**Y073**	55	31.03.2011	large	spraint	non-exposed	>4	129	145	137	137	211	211	141	141	140	142	172	196	150	150	F
**Y075**	76	31.03.2011	medium	spraint	non-exposed	>4	129	145	135	137	211	211	135	135	140	142	176	176	150	150	F
**Y083**	1	31.03.2011	medium	jelly	actively exposed	>4	129	129	135	137	211	211	135	135	140	142	172	180	150	150	F
**Y090**	64	31.03.2011	medium	mucous spraint	non-exposed	3-4	129	129	135	139	211	211	135	135	140	142	172	196	150	150	M
**Y093**	44	31.03.2011	medium	spraint	non-exposed	>4	129	129	137	139	207	211	135	141	140	142	180	180	146	150	F
**Y096**	67	31.03.2011	medium	jelly	non-exposed	>4	129	129	137	141	211	211	135	151	140	142	180	196	146	150	M
**Y097**	21	31.03.2011	small	mucous spraint	actively exposed	>4	145	145	135	141	211	211	135	141	140	142	176	196	150	150	M
**Y101**	69	31.03.2011	large	mucous spraint	actively exposed	1-2	129	145	139	139	211	211	135	141	140	140	172	176	146	150	F
**Y103**	65	31.03.2011	small	jelly	passively exposed	3-4	129	129	137	137	211	211	135	135	140	142	172	172	150	150	M
**Y117**	67	31.03.2011	medium	jelly	non-exposed	1-2	129	129	137	141	211	211	135	151	140	142	180	196	146	150	M
**Y123**	59	31.03.2011	large	spraint	passively exposed	>4	129	129	137	139	211	215	135	141	140	142	172	180	150	150	M
**Y136**	56	31.03.2011	medium	jelly	actively exposed	1-2	129	145	137	139	211	211	141	151	140	142	180	196	150	150	M
**Y137**	56	31.03.2011	small	jelly	non-exposed	1-2	129	145	137	139	211	211	141	151	140	142	180	196	150	150	M

**Y142**	70	31.03.2011	medium	spraint	actively exposed	>4	129	145	139	141	211	211	135	141	140	142	180	180	150	150	F
**Y147**	56	31.03.2011	small	jelly	actively exposed	1-2	129	145	137	139	211	211	141	151	140	142	180	196	150	150	M
**Y148**	69	31.03.2011	small	mucous spraint	actively exposed	>4	129	145	139	139	211	211	135	141	140	140	172	176	146	150	F
**Y150**	75	31.03.2011	large	mucous spraint	actively exposed	1-2	129	145	135	139	211	211	135	141	140	140	172	176	146	150	M
**Z015**	42	01.04.2011	large	spraint	passively exposed	3-4	129	139	135	139	211	211	135	141	140	142	176	180	150	150	M
**Z027**	57	01.04.2011	large	mucous spraint	actively exposed	>4	129	147	139	139	211	211	135	135	140	142	172	172	146	150	F
**Z065**	64	01.04.2011	large	mucous spraint	non-exposed	>4	129	129	135	139	211	211	135	135	140	142	172	196	150	150	M
**Z070**	65	01.04.2011	medium	spraint	non-exposed	3-4	129	129	137	137	211	211	135	135	140	142	172	172	150	150	M
**Z073**	58	01.04.2011	medium	mucous spraint	non-exposed	>4	129	145	135	137	211	211	135	135	140	142	176	180	150	150	F
**Z080**	44	01.04.2011	small	spraint	non-exposed	1-2	129	129	137	139	207	211	135	141	140	142	180	180	146	150	F
**Z083**	73	01.04.2011	medium	spraint	non-exposed	>4	129	139	139	139	211	211	135	141	140	142	176	180	150	150	M
**Z093**	44	01.04.2011	medium	mucous spraint	non-exposed	3-4	129	129	137	139	207	211	135	141	140	142	180	180	146	150	F
**Z097**	67	01.04.2011	large	mucous spraint	actively exposed	3-4	129	129	137	141	211	211	135	151	140	142	180	196	146	150	M
**Z102**	57	01.04.2011	large	spraint	non-exposed	3-4	129	147	139	139	211	211	135	135	140	142	172	172	146	150	F
**Z103**	53	01.04.2011	medium	mucous spraint	passively exposed	3-4	145	145	135	137	211	211	135	141	140	142	176	180	150	150	F
**Z106**	55	01.04.2011	large	spraint	actively exposed	1-2	129	145	137	137	211	211	141	141	140	142	172	196	150	150	F
**Z109**	57	01.04.2011	large	mucous spraint	actively exposed	>4	129	147	139	139	211	211	135	135	140	142	172	172	146	150	F
**Z110**	71	01.04.2011	large	mucous spraint	actively exposed	3-4	129	147	135	139	211	211	135	135	140	140	172	180	146	150	F
**Z122**	56	01.04.2011	small	jelly	passively exposed	3-4	129	145	137	139	211	211	141	151	140	142	180	196	150	150	M
**Z125**	69	01.04.2011	small	spraint	actively exposed	3-4	129	145	139	139	211	211	135	141	140	140	172	176	146	150	F
**Z136**	67	01.04.2011	small	mucous spraint	passively exposed	3-4	129	129	137	141	211	211	135	151	140	142	180	196	146	150	M
**Z137**	67	01.04.2011	large	mucous spraint	non-exposed	3-4	129	129	137	141	211	211	135	151	140	142	180	196	146	150	M
**Z140**	67	01.04.2011	large	mucous spraint	non-exposed	1-2	129	129	137	141	211	211	135	151	140	142	180	196	146	150	M
**ZX1**	57	01.04.2011	small	spraint	actively exposed	>4	129	147	139	139	211	211	135	135	140	142	172	172	146	150	F
**ZX2**	57	01.04.2011	medium	mucous spraint	actively exposed	>4	129	147	139	139	211	211	135	135	140	142	172	172	146	150	F
**ZX3**	57	01.04.2011	large	spraint	actively exposed	>4	129	147	139	139	211	211	135	135	140	142	172	172	146	150	F
